# Integrative glycomic analysis reveals the crucial role of protein glycosylation in fungal pathogenesis

**DOI:** 10.1371/journal.ppat.1013325

**Published:** 2025-07-07

**Authors:** Heeji Moon, Eun Jung Thak, Yejin Choi, Sieun Kim, Jiyeun Park, Nahyun Lee, Soobin Shin, Hosung Jeon, Jessica Winarto, Soyoung Choi, Ji Young Shin, Jung-Eun Kim, Dae-Geun Song, Hun Kim, Gyung Ja Choi, Hyun Ah Kang, Hokyoung Son

**Affiliations:** 1 Department of Agricultural Biotechnology, Seoul National University, Seoul, Republic of Korea; 2 Department of Life Science, Chung-Ang University, Seoul, Republic of Korea; 3 College of Pharmacy, Dongduk Women’s University, Seoul, South Korea; 4 Horticultural and Herbal Crop Environment Division, National Institute of Horticultural and Herbal Science, Wanju, South Korea; 5 Institute for Plant Sciences, University of Cologne, Cologne, Germany; 6 Center for Natural Product Systems Biology, Korea Institute of Science and Technology (KIST) Gangneung Institute of Natural Products, Gangneung, Republic of Korea; 7 Division of Bioresources Bank, Honam National Institute of Biological Resources, Mokpo, Republic of Korea; 8 Research Institute of Climate Change and Agriculture, National Institute of Horticultural and Herbal Science, Rural Development Administration, Jeju, Republic of Korea; 9 Natural Product Applied Science, KIST School, University of Science and Technology, Gangneung, Republic of Korea; 10 Therapeutic & Biotechnology Division, Center for Eco-friendly New Materials, Korea Research Institute of Chemical Technology, Daejeon, Republic of Korea,; 11 Research Institute of Agriculture and Life Sciences, Seoul National University, Seoul, Republic of Korea; 12 Plant Genomics and Breeding Institute, Seoul National University, Seoul, Republic of Korea; 13 Plant Health Center, Seoul National University, Seoul, Republic of Korea; Purdue University, UNITED STATES OF AMERICA

## Abstract

Protein glycosylation, a co- and post-translational modification that enhances the functional diversity of the proteome, contributes to various molecular and cellular functions by transferring different polysaccharides onto proteins. During the last decade, the role of glycosylation in plant pathogenic fungi has received significant attention, and glycoproteins are expected to play essential roles in various biological processes including pathogenicity. However, the comprehensive functional genetic analyses for protein glycosylation pathways and glycan structures of phytopathogenic fungi are still largely unknown. Here, we investigated the role of protein glycosylation in *Fusarium graminearum* by identifying 65 putative genes involved in protein glycosylation and characterizing their functions. Through cell wall component profiling and HPLC analysis, we characterized the overall *N*- and *O*-glycan structures in *F. graminearum* and found that deletion of *ALG3* and *ALG12* led to truncated core *N*-glycan structures. Quantitative proteomics analysis revealed that the truncated core *N*-glycans, generated by the loss of two key enzymes in the initial core *N*-glycosylation pathway, Alg3 and Alg12, affected a wide range of glycoproteins—including transcription factors, phosphatases, kinases, peroxidases, and other proteins involved in various biological processes—ultimately impacting the virulence of *F. graminearum*. This study elucidates the complex roles of glycosylation, highlighting the connections among genes involved in the protein glycosylation pathway, glycans, and glycoproteins in regulating the general biology and pathogenicity of *F. graminearum*. It also would be the fungal glycobiology study initiative.

## Author summary

Protein glycosylation enhances proteome diversity by transferring various polysaccharides onto proteins and contributes to many biological processes. However, comprehensive functional genetic analyses of protein glycosylation pathways and glycan structures in phytopathogenic fungi remains poorly understood. In this study, we identified 65 genes involved in protein glycosylation in *F. graminearum* and investigated their biological roles. We further characterized the overall *N*- and *O*-glycan structures, establishing a framework for future studies on glycan profiling in *F. graminearum.* Deletion of two key glycosylation genes, *ALG3* and *ALG12*, resulted in truncated core *N*-glycans, which affected numerous glycoproteins involved in various cellular functions and led to reduced fungal virulence. Our findings highlight the importance of glycosylation in fungal biology and pathogenicity, providing a foundation for future research in fungal glycobiology and the development of novel disease control strategies.

## Introduction

Protein glycosylation is a prevalent post-translational modification crucial for protein folding, stability, quality control, transport, and localization [1,2]. This process entails the addition of glycans to specific amino acid residues: asparagine for *N*-glycosylation [3] and hydroxylysine, hydroxyproline, serine, or threonine for *O*-glycosylation [4,5]. The coordinated action of numerous genes encoding glycosyltransferases, glycosidases, and other enzymes orchestrates protein glycosylation in the endoplasmic reticulum (ER), where glycan chains are synthesized and transferred to proteins, and Golgi apparatus, where glycan chains are subjected to further processing. These pathways of protein glycosylation have been extensively elucidated in eukaryotic model organisms such as *Saccharomyces cerevisiae* [6]*, Schizosaccharomyces pombe* [7,8]*, Cryptococcus neoformans* [9]*,* and mammalian systems [10].

Numerous studies underscore the significance of protein glycosylation in diverse biological processes across species. In humans, alterations in glycosylation can affect inflammatory responses, facilitate viral immune escape, promote cancer cell metastasis, or regulate apoptosis [11]. Glycoengineering in various yeasts has been conducted to enable the production of therapeutic proteins with human-compatible protein glycosylation [12]. In *S. cerevisiae,* a strain with abolished mannose phosphorylation showed it’s potential for producing humanized proteins [13]. Glycoengineered *Pichia pastoris* generates a homogeneous antibody through transglycosylation using glycans with a stable leaving group [14]. In *C. neoformans*, one of the critical human pathogenic fungi, core *N*-glycan structures are essential for pathogenicity by modulating host cell death [15], while *O*-linked mannosylation plays a critical role in cell wall integrity signaling and host cell interactions [16].

In the past decade, the role of protein glycosylation in plant pathogenic fungi has garnered significant attention, with glycoproteins expected to be pivotal in pathobiological processes [17]. Reverse and forward genetic approaches have uncovered that several glycosyltransferases and glucosidases affect fungal growth and virulence in *Magnaporthe oryzae*, *Glomerella graminicola*, *Botrytis cinerea*, *Ustilago maydis*, and *Zymoseptoria tritici* [18–22]. Furthermore, comparative glycoproteomic studies have identified various glycoproteins involved in protein modification, quality control, and virulence in *M. oryzae*, *B. cinerea*, *G. graminicola*, *F. oxysporum*, and *F. graminearum* [21,23–26]. Despite efforts to analyze the functions of individual genes and glycoproteins, comprehensive functional genetic analyses of protein glycosylation and structural profiling of *N*- and *O*-glycans attached to glycoproteins remain unexplored in plant pathogenic fungi.

The filamentous ascomycete fungus *F. graminearum* is a significant plant pathogen that causes Fusarium head blight in wheat and barley and ear rot in maize worldwide [27–29]. *F. graminearum* infections lead to substantial yield losses and mycotoxin contamination, such as deoxynivalenol (DON) and zearalenone (ZEA), posing threats to human and animal health [30–32]. Besides its economic impact, this fungus also serves as a model organism for molecular and genomic studies of plant pathogenic fungi because of its well-established genetic manipulation techniques and wide-ranging ongoing research into its genomics, transcriptomics, proteomics, and reverse genetics [33–35]. Particularly, extensive large-scale reverse genetic approaches have been undertaken over decades to elucidate the molecular mechanisms underlying various biological processes and virulence of this fungus [36–44]. However, a comprehensive understanding of the pathobiological networks of *F. graminearum* remains elusive, primarily due to the limited characterization of post-translational modifications, which play a crucial role in increasing the functional diversity of the proteome.

In this study, we systematically profiled the glycomics of *F. graminearum* by integrating three multidisciplinary datasets, including phenome data obtained from a genome-wide deletion mutant library comprising 65 putative glycosylation-related genes, profiles of *N-* and *O*-glycan structures, and comparative glycoproteomic data. Collectively, these integrated datasets unveiled a trend where the severity of phenotypic traits diminished toward the late stages of the protein glycosylation process. Furthermore, our analysis pinpointed Alg12 and Alg3 orthologs, implicated in the initial core glycan structures, as not only reducing the total glycoprotein pool but also affecting the *N*-glycosylation of cell wall glycoproteins, which have been previously associated with virulence. This study sheds light on the critical role of protein glycosylation in various cellular processes, including fungal pathogenesis, marking it as the inaugural systematic glycomics investigation in plant pathogenic fungi.

## Materials and methods

### Fungal strain and culture conditions

The *F. graminearum* wild-type strain Z-3639 [45] was used in all experiments. The culture media were prepared following the Fusarium laboratory manual [46]. All of the wild-type strain and transformants used in this study were grown in CM agar plates at 25 °C and stored as mycelial suspensions in 20% glycerol at −80 °C. Conidia of all strains were induced either in carboxymethyl cellulose (CMC) medium or on yeast malt agar (YMA). To obtain mycelia, conidial suspensions of the wild-type strains were inoculated in liquid CM (5 × 10^6^ conidia/mL) and mycelia were harvested after 24 h of incubation on a rotary shaker (200 rpm).

### Nucleic acid manipulations, Southern blotting, and PCR

For genomic DNA isolation, each strain was cultured in 5 ml of CM for 3 days in a rotary shaker at 200 rpm, and genomic DNA was extracted according to the Fusarium laboratory manual [46]. Restriction endonuclease digestion and agarose gel electrophoresis were performed following standard protocols. [47] Southern blot hybridization was performed with the North2South Biotin Random Prime Labeling Kit and the North2South Chemiluminescent Hybridization and Detection Kit (Thermo Fisher Scientific, Waltham, MA, USA). The PCR primers used in this study (S1 Table) were synthesized by an oligonucleotide synthesis facility (Bioneer, Daejeon, Korea).

### Genetic manipulations and fungal transformations

The double-joint (DJ) PCR method [48] was used to generate the fusion PCR products that were required for the gene deletion, complementation, and overexpression. The fungal transformation was performed as previously described [49].

To construct the deletion mutants, the 5′ and 3′ flanking regions of the target genes were amplified from the genomic DNA, and the Geneticin resistance gene cassette (*GEN*) was amplified from pII99 [50]. Three fragments were fused via the DJ PCR method, and the final constructs were amplified using the nested primers. The resulting amplicons were transformed into the fungal wild-type protoplasts, using PEG-mediated transformation [51]. The mutant was confirmed via Southern hybridization with a flanking region probe.

### Vegetative growth, conidiation, and sexual development

Radial growth and colony morphology were assayed on CM and MM 4–5 days after inoculation. For the conidiation assays, a fresh mycelial plug from CM was inoculated in 5 mL of CMC for 5 days on a rotary shaker (200 rpm). The number of conidia was measured with a hemacytometer.

For sexual development, fungal strains were grown on carrot agar for 5 days, and aerial mycelia were removed with 0.4 mL of a sterile 2.5% Tween 60 solution. The plates were then incubated under near-UV light (wavelength: 365 nm; Sankyo Denki, Tokyo, Japan). The number of perithecia, maturation, ascospore morphology, and ascospore discharge were observed after 7–9 days.

For outcrosses, the female strain was fertilized with 1 mL of a conidial suspension from the male strain 5 days after inoculation on carrot agar.

### Cellophane membrane penetration assay

To examine the penetration ability, each strain was inoculated on cellophane-overlaid CM. Cellophane membranes were removed after 36 and 48 h post-inoculation, respectively, and the resulting media were observed after removal. All penetration experiments were conducted three times.

### Stress response

The stress responses were evaluated by assaying vegetative growth on CM supplemented with various stress agents: oxidative stress (7 mM hydrogen peroxide and 38 μM menadione), osmotic stress (1 M NaCl, 1 M KCl, 1.5 M sorbitol), ionic stress (5 mM FeSO_4_), pH stress (pH 4 and pH 11), cell wall stress (400 mg/L Congo red and 100 mg/L sodium dodecyl sulfate), DNA synthesis inhibition (8.6 mg/L iprodione), the inhibition of mitogen activated protein kinase (0.023 mg/L fludioxonil), the inhibition of meiosis and intracellular transportation (0.1 mg/L benomyl), the inhibition of glycan synthesis (0.2 μg/ml caspofungin, 0.02 μg/ml micafungin, 0.03 μg/ml anidulafungin, and 2.5 μg/ml dimethomorph), the ER stress agents (10 mM DTT and 2.5 μg/ml tunicamycin) and azole fungicide (0.1 mg/L tebuconazole) [36].

### Virulence test and mycotoxin analysis

The point inoculation method was performed to assay fungal virulence as previously described [49]. Conidia were harvested from CMC cultures, and 10 μL of each suspension (10^5^ conidia/mL) was injected into the center spikelet of a wheat head (cultivar: Eunpamil). The inoculated wheat plants were incubated in a humid chamber for 3 days and grown in a greenhouse for an additional 18 days. The number of diseased spikelets was measured 21 days after inoculation.

The DON and ZEA extraction was performed as previously described [52]. A fresh mycelial plug was inoculated on 1.5 g of rice substrate for 3 weeks. The rice culture was harvested, ground, and mixed vigorously with 6 mL of 84% acetonitrile for 30 min. After phase separation, the upper phase was filtered through a 0.45 μm syringe filter. Reverse-phase HPLC on a Prominence HPLC system (Shimadzu, Kyoto, Japan) with a C18 column was used for the analysis, with a simple modification of the previous methods [53,54]. For ZEA detection, the mobile phase was 70% aqueous methanol, and the flow rate was 1 mL min^−1^. For DON detection, the mobile phase was 10% aqueous acetonitrile (ACN), and the flow rate was 1 mL min^−1^. A gradient elution program was applied as follows: after 10% ACN was maintained for 11 min, it was linearly increased to 30% ACN at 12 min. It was then linearly decreased from 30% ACN at 12 min to 10% ACN at 18 min. Subsequently, 10% ACN was held for 17 min for the reequilibration of the column before the injection of the next sample, giving a total run time of 35 min. Diode-array detection was applied (ZEA and DON were detected at a wavelength of 235 nm).

### HPLC analysis of *N&O*-linked glycans from cell wall mannoproteins

In our study, we performed HPLC-based glycan profiling to obtain information on structures of glycans, which are separated based on their physical and chemical properties. The retention times of glycan peaks were compared with those of standard glycans.

cwMPs were isolated from *F*. *graminearum* mycelia and conidia as described previously [9,55,56]. *N*-linked glycans were released from the purified cwMPs using PNGase F (New England Biolabs, Ipswich, MA, USA) and were purified over a Carbograph Extract-Clean (Grace Davison Discovery Science, Bannockburn, IL, USA) column. The *N*-glycans were labeled with 2-aminobenzoic acid (2-AA; MilliporeSigma, Burlington, MA, USA) and purified using a Cyano Base cartridge (Bond Elut-CN-E; Agilent, Santa Clara, CA, USA) (100 mg) to remove excess 2-AA. 2-AA-labeled oligosaccharides were analyzed with a Waters 2690 HPLC system and a 2475 fluorescence detector with excitation and emission wavelengths of 360 nm and 425 nm, respectively. Data were collected using Empower 2 software (Waters, Milford, MA, USA).

For release of the *O*-glycans, the completely dried cwMPs (50 μg) were resuspended in 100 μL of hydrazine monohydrate (Tokyo Chemical Industry, Tyoko, Japan), and the mixture was incubated at 60 °C for 4–6 h. The reactants were dried to remove the hydrazine monohydrate, and the pellets were dissolved in 100 μL of saturated NaHCO_3_ (Sigma, Burlington, MA, USA), mixed with 10 μL of (CH_3_CO)_2_O, and incubated on ice for 30 min without shaking. The *O*-glycans were purified by using Dowex 50WX8–400 resins (H^+^ form; Sigma), and the isolated *O*-glycans were labeled with 2-aminobenzoic acid (2-AA; Sigma) and purified using a cyano base cartridge (Bond Elut-CN-E; Agilent) (100 mg). The HPLC analysis of the 2-AA-labeled *O*-glycan was conducted on a TSKgel Amide-80 column (0.46 by 25 cm, 5 μm; Tosoh, Tokyo, Japan) at a flow rate of 1.0 mL/min. 2-AA-Oligosaccharides were detected with a 2475 fluorescence detector (Waters) at excitation and emission wavelengths of 360 and 425 nm, respectively. Data were collected using Empower 2 chromatography data software (Waters).

### Microscopic observation

Microscopic observation was performed with a DM6 B microscope (Leica Microsystems, Wetzlar, Germany) equipped with a Leica DMC6200 camera using an L5 fluorescence filter (excitation 480/40 nm; emission 527/30 nm) for GFP, an N2.1 filter (excitation 515–60 nm; emission 590 nm) for RFP, and a DAP filter (excitation 355–425 nm; emission 470 nm) for UV and violet fluorescent.

### Western blotting

Conidial suspensions were inoculated in CM at 5 × 10^6^ conidia per milliliter. Mycelia were harvested 24 h after incubation on a rotary shaker (200 rpm) and washed twice with distilled water. For total protein extraction, fresh mycelia were ground in liquid nitrogen and re-suspended in 1 mL extraction buffer containing protease inhibitors. Lysates were sonicated and centrifuged at 13,000 rpm for 20 min. The resulting supernatants were quantified with Pierce 660 nm Protein Assay Reagent (Thermo Scientific,  San Jose, CA) and were used for western blot analysis. To detect the overall protein *N*-glycosylation level, total proteins were mixed with SDS-PAGE loading buffer, denatured at 95 °C for 10 min, and then subjected to 8% SDS polyacrylamide gels and transferred to nitrocellulose membranes using the Trans-Blot Turbo Transfer System (Bio-Rad, Hercules, CA, USA). Glycoproteins were detected with a Con A-HRP (1:10,000) as an antibody and photographed using a Chemi-Doc imaging system (Bio-Rad). Relative protein abundance was calculated using Image Lab 4.1 software (Bio-Rad) by comparing the adjusted total lane volume of Con A-HRP-detected glycoproteins with that of the corresponding stain-free gel-detected total protein bands.

To analyze *N*-glycosylation level of each target glycoproteins from wild type and deletion mutant strains, each target glycoproteins were detected using an anti-GFP antibody (1:5,000; Abcam, Cambridge, UK) as the primary antibody. Protein samples were digested with PNGaseF (P0704; New England Biolabs) for 1 h to cleave between the innermost GlcNAc and asparagine residues of high-mannose, hybrid, and complex oligosaccharides. The digested samples were then mixed with the loading buffer. A theoretically smaller protein band of digested target was detected by immunoblot analysis as previously described [20].

### Cell wall components analysis

The cell wall isolation and purification were prepared as described previously [57]. Conidia and mycelium, harvested by vacuum filtration and immediately frozen in liquid nitrogen, were ground into a fine powder under liquid nitrogen. This powder was then transferred to a 2.0 mL screw cap tube (Thermo Fisher Scientific) and disrupted with silica beads (BioSpec Product, Bartlesville, OK) using a Fastprep-24 5G machine (MP Biomedicals, Santa Ana, California, USA). This procedure was carried out until complete cell breakage was achieved (room temperature for eight periods of 1 min on ice), as confirmed by examination under a phase-contrast microscope and by the failure of extracts to produce viable colonies when they were plated on CM agar medium. Cell lysates were separated into a cell wall fraction (pellet) and a soluble cytoplasmic fraction (supernatant) by centrifugation at 1000x*g* and 4 °C for 10 min. To reduce cytoplasmic contaminations, washing procedures were as follows: First, the cell wall fraction was washed three times in 1 × PBS. Next, the cell wall pellet was washed three times each in 5, 2.5, and 1.25% sodium chloride (NaCl) solution. Finally, native cell walls were obtained by further incubating overnight in 0.1 M sodium carbonate (Na_2_CO_3_) solution and rinsed in 1 × PBS.

For monosaccharide analysis, 100 mg of cell wall material was hydrolyzed under different conditions: in 6 N HCl at 100 °C for 4 h for amino sugar (glucosamine and galactosamine) analysis, in 2 M trifluoroacetic acid at 100 °C for 4 h for neutral sugar (fucose, galactose, glucose, and mannose) analysis, and in 0.1 N HCl at 80 °C for 1 h for sialic acid analysis.

The sialic acid, neutral and amino sugars were separated and quantitated on a CarboPac PA10 column (4.0 x 250 mm, Dionex Co., Sunnyvale, CA, USA) and CarboPac PA1 column (4.5 x 250 mm), with an ICS-5000 with electrochemical detection using a CarboPac PA1 (4.5 x 50 mm) and CarboPac PA10 (4.0 x 50 mm) cartridge. 16 mM NaOH was used as an eluant at a flow rate of 1.0 ml/min. Duplicated data was analyzed using PeakNet on-line software. The analysis was carried out at the Korea Basic Science Institute (KBSI, located in Ochang, South Korea).

### Preparation of glycoprotein samples for proteomic analysis

The glycoproteins were obtained as described previously with a slight modification [23,24]. The total proteins were dissolved with 10 mL of Con A binding buffer (20 mM Tris-HCl, pH 7.4, 0.5 M NaCl, 1 mM CaCl_2_, 1 mM MnCl_2_) and incubated with 1 mL of Con A-Sepharose beads (GE Healthcare, Chicago, IL) in a column for 2 h with slow rotation. The beads were then washed with 10 mL of Con A binding buffer, and glycoproteins were eluted by the addition of 5 mL of 0.5 M methyl-*α*-d-mannopyranoside and 0.5 M methyl *α*-glucopyranoside. The whole cell extracts (total proteins) and glycoproteins were quantified with Pierce 660 nm Protein Assay Reagent (Thermo Scientific). After boiling sample, the denatured proteins were separated on SDS polyacrylamide gels and stained with Coomassie Brilliant Blue. Bands of interest were excised, and trypsin digested.

### Tandem mass spectrometry analysis

Tryptic peptides were analyzed using a Q Exactive system equipped with EASY-nLC-1000 nano flow liquid chromatography system (Thermo Scientific). Samples were trapped in an Acclaim PepMap 100 C18 column (75 µm × 2 cm, Thermo Scientific) and separated on an EASY-Spray column (75 µm × 15 cm, particle size ≤3 m, pore size 100 Å, PepMap RSLC C18, Thermo Scientific) using a 90 min gradient of solvent A and B (0.1% FA in ACN). The gradient included 5–40% solvent B at a flow rate of 300 nL/min and a column temperature of 35 °C. The eluted peptides were ionized at 2 kV. A full MS scan was performed from 400 to 2000 m/z with a full width a (FWHM) resolution of 70,000, followed by a data-dependent HCD MS/MS scan of the top 10 ions. Parameters used for MS/MS scan were resolution = 17,500, loop count = 10 (Top 10), isolation window = m/z 2.0, normalized collision energy = 27.

### Data analysis

The raw MS spectra files were searched against the *F. graminearum* protein database (47,879 entries downloaded from UniProtKB on Apr. 4^th^, 2024) using Proteome Discoverer (v2.4.1.15; Thermo Fisher Scientific). The search settings were as follows: Sequest HT search engine, trypsin (full), maximum two missed cleavage sites, MS tolerance (10 ppm), MS/MS tolerance 0.02 Da, carbamidomethylation (C) for fixed modification, and oxidation (M) for variable modification. Peptide/protein identification and quantitation were conducted on Scaffold (v5.0.1, Proteome Software Inc.) with a SEQUEST XCorr threshold of at least 1.8 for charge 1, 2.5 for charge 2, and 3.5 for charges 3 and 4. Total spectrum count was used for semiquantitative analysis. For normalization, the endogenous housekeeping protein GAPDH was used as an internal control. The mass spectrometry proteomics data have been deposited to the ProteomeXchange Consortium via the PRIDE partner repository under the accession number PXD063761.

### Bioinformatic analysis

Gene Ontology (GO) analysis was performed using the REVIGO in FungiDB [58] database and ClueGO [59] software tool. The Kyoto Encyclopedia of Genes and Genomes (KEGG) database (http://geneontology.org/) was used to annotate the pathways enriched in each protein group. These results were visualized using the R program.

Glycoproteins that were differentially regulated by Alg3 (Fg26583) and Alg12 (Fg03053) were selected to establish the PPI network. PPI network was visualized using Cytoscape software (version 3.10.0) performed using the psych R-package with standard settings. The principal component analysis (PCA) was performed using the psych R-package with standard settings. Spearman’s rank correlation analysis was performed using the psych R package, with significance determined at P < 0.05 (two-tailed).

## Results

### Identification of protein glycosylation-related genes in *F. graminearum*

**Fig 1:**
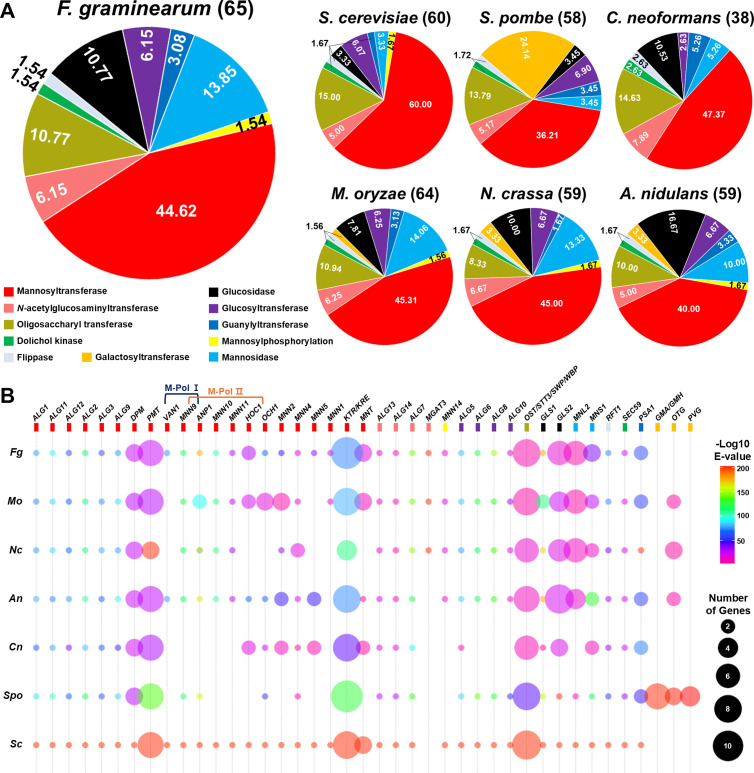
Comparative analysis of protein glycosylation-related genes among fungal species. **(a)** Pie chart indicating the classification and distribution of putative protein glycosylation-related genes in the fungal species, *F. graminearum, S. cerevisiae*, *S. pombe*, *C. neoformans, N. crassa, M. oryzae,* and *A. nidulans.* The genes were classified based on both their function and the type of oligosaccharides they transfer. **(b)** Dot plot shows the homologues of genes related to protein glycosylation across the different fungal species. The number of genes in each species is represented by the areas of the circles. The identity of protein sequence values in each species were represented based on *S. cerevisiae* and *S. pombe*. Genes are categorized and represented with distinct colors based on their respective functions, employing the same strategies as illustrated in Fig 1a.

To identify genes potentially involved in protein glycosylation in *F. graminearum*, we employed a multi-step approach. Initially, we retrieved genes from three Kyoto Encyclopedia of Genes and Genomes (KEGG) pathway databases (map00510, *N*-Glycan biosynthesis; map00513, Various types of *N*-glycan biosynthesis; map00515, Mannose type *O*-glycan biosynthesis; map00514, Other types of *O*-glycan biosynthesis). Additionally, leveraging the well-established protein glycosylation pathways in *S. cerevisiae* and *S. pombe*, we supplemented our search with protein glycosylation-related genes from the *Saccharomyces* Genome Database (SGD) [60] and PomBase [61]. Utilizing BLASTp, we compared the sequences of *S. cerevisiae* and *S. pombe* genes to identify orthologs in the genomes of various fungal species, including *F. graminearum.* Through these analyses, we identified 65 putative protein glycosylation-involved genes in *F. graminearum* (Fig 1a, b and S2 Table). The genes were further classified based on both their function and the type of oligosaccharides they transfer.

Our investigation yielded several noteworthy observations. First, the proportion of classified genes varied among species (Fig 1a). Mannosidases were notably more abundant in filamentous fungi than in yeast-form fungi, whereas glucosidases were less represented in *S. cerevisiae* and *S. pombe* compared to other fungal species. Additionally, mannosyltransferases were significantly more prevalent in *S. cerevisiae* compared to other fungal species. Interestingly, the overall ratio of classified genes was similar in filamentous fungi, whereas it differed considerably in yeast-form fungi. In the case of *C. neoformans*, its overall ratio was more similar to that of filamentous fungi, except for glucosidases. Second, our analysis revealed the presence of certain glycosyltransferases that are species-specific (Fig 1b). For example, galactosyltransferase only existed in *M. oryzae*, *N. crassa*, *A. nidulans*, and *S. pombe* but not in other fungi; *β*-1,4-mannosyl-glycoprotein 4-*β*-*N* acetylglucosaminyltransferase (*MGAT3*) was found only in filamentous fungi; the number of genes encoding M-pol I (*VAN1* and *MNN9*) and M-pol II (*MNN9*, *ANP1*, *MNN10*, *MNN11*, and *OCH1*) subunits were varied across species. Third, the overall protein sequence similarity (E-value) was comparable across species. These findings highlight the diversity and specificity of protein glycosylation-related genes across fungal species, suggesting potential targets for future comparative functional studies.

### Construction of the *F. graminearum* mutant library involved in protein glycosylation

To delve into the biological functions of the 65 genes involved in protein glycosylation in *F. graminearum,* we systematically constructed deletion mutants for each of the *N*- and *O*-glycosylation-related genes, followed by comprehensive functional characterization. With the exception of *SEC59* (*Fg00930*), which was generated in a previous study [44], we generated gene replacement constructs for all other glycosylation-related genes using the split-marker approach. These constructs were then transformed into protoplasts of the *F. graminearum* wild-type strain Z-3639. Through homologous recombination, we successfully deleted 52 out of 65 putative glycosylation-related genes (S3 Table, Fig A in S1 Text).

For the remaining 13 putative glycosylation-related genes (S3 Table), despite screening over dozens of transformants from at least three independent transformation experiments, we were unable to identify knockout mutants, indicating that the deletion of these genes might be lethal. Notably, ten of these genes have orthologs that are essential in both *S. cerevisiae* and *S. pombe*, indicating their critical functions across fungal species. The remaining five genes showed species-specific lethality. Intriguingly, ten genes exhibited lethality exclusively in *S. cerevisiae* and *S. pombe*, while they remained non-lethal in *F. graminearum*. Among the ten genes, only three were found to have paralogs in filamentous fungi (S2 Table). For example, in the case of *CWH41/MOGS* (*Fg17395*), two paralogous genes (*MGG_04045* and *MGG_09721*) were identified in *M. oryzae*. Notably, *MGG_04045* has been previously characterized as *GLS1*, which was shown to be non-essential [24]. For *PSA1* (*Fg17687*), two homologous copies are present even within *F. graminearum* itself (including *Fg10789*). Similarly, *M. oryzae* possesses two *PSA1* homologs (*MGG_01288* and MGG_05936), and *A. nidulans* has two (*AN1911* and *AN5586*). *N. crassa* is unique among the species examined in containing only a single *PSA* ortholog (*NCU06003*). Lastly, for *STT3* (*Fg01117*), two copies were identified in *M. oryzae* (*MGG_02821* and *MGG_04773*). These observations suggest that the presence of paralogs in filamentous fungi may contribute to functional compensation, potentially explaining why some of these genes appear non-essential in *F. graminearum*. Among the remaining seven genes, *ALG2* (*Fg04001*), *RFT1* (*Fg06329*), and *ALG11* (*Fg20645*) exhibited severe growth defects upon deletion. These genes may thus be considered as quasi-essential or fitness-critical, as their essentiality differs across species, but they likely play key roles in cellular viability and growth. These findings imply the presence of functional divergence in certain glycosylation genes among different fungal species or the presence of functionally redundant glycosylation genes within *F. graminearum.*

### Profiling the collective phenotypic traits of the *F. graminearum* mutant library

Given the variation in the presence and proportion of protein glycosylation-related genes among fungal species, we compared our mutant phenotypic data, particularly focusing on growth and virulence, with relevant studies on glycosylation in other species [15,17,62–65] (S3 Table and Fig B in S1 Text). While studies on protein glycosylation-related genes in fungal species like *M. oryzae*, *G. graminicola*, and *C. neoformans* are less abundant compared to well-studied model organisms such as *S. cerevisiae*, *S. pombe*, and *C. albicans,* our comparative analysis revealed both unique and conserved functions in regulating growth and virulence across fungal species.

**Fig 2:**
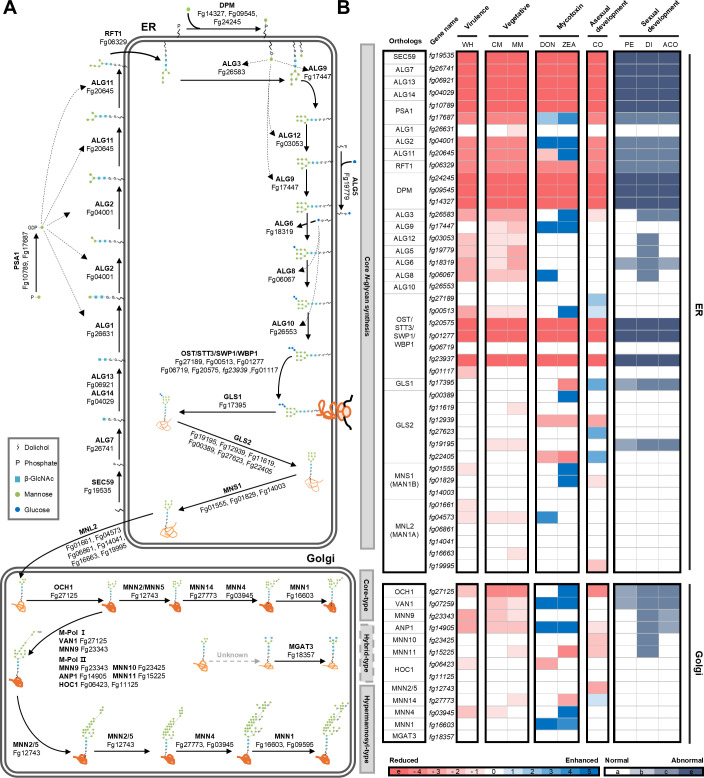
Profiling overall protein *N*-glycosylation pathway genes in *F. graminearum.* **(a)** Schematic diagram of the protein *N*-glycosylation pathway in *F. graminearum*. The sequences of all genes participating in *N*-glycan biosynthesis from the budding yeast *S. cerevisiae, S. pombe* and *Candida albicans* were used for BLASTp searches to identify their orthologs in the genome of *F. graminearum*. **(b)** Phenotypic traits of genes involved in protein *N*-glycosylation at the ER (endoplasmic reticulum) and Golgi (golgi apparatus) were examined. virulence, vegetative growth, mycotoxin, and asexual development were categorized as continuous phenotypic traits and scored on a 11-point scale (e: essential, -4: reduced rate between 0 and 0.29, -3: reduced rate between 0.3 and 0.49, -2: reduced rate between 0.5 and 0.69, -1: reduced rate between 0.7 and 0.89, 0: no difference compared with wild-type, + 1: enhanced rate between 1.10 and 0.29, + 2: enhanced rate between 1.30 and 1.49, + 3: enhanced rate between 1.50 and 1.69, +4: enhanced rate between 1.70 and 1.89, +5: enhanced rate over 1.90). Sexual developments were categorized as discrete phenotypic traits and scored on a 4-point scale (a: no difference compared with wild-type, b: decreased number of perithecia and ascospore or delayed perithecia maturation or decreased number of discharged ascospores, c: no perithecia and ascospore formation or no ascospore discharge, e: essential genes). More than three biologically independent experiments were performed for each phenotypic trait. Abbreviations: WH wheat head, CM complete media, MM minimal media, DON deoxynivalenol, ZEA zearalenone, CO conidia, PE perithecia, DI discharge, ACO ascospore.

**Fig 3:**
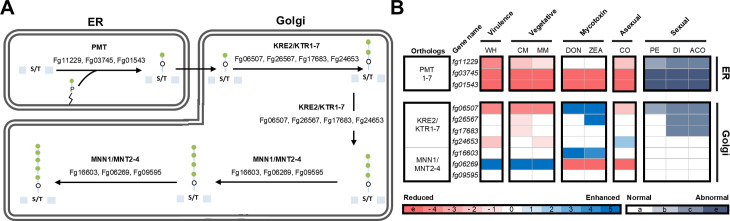
Profiling overall protein *O*-glycosylation pathway genes in *F. graminearum.* **(a)** Schematic diagram of the protein *O*-glycosylation pathway in *F. graminearum*. The sequences of all genes participating in *O*-glycan biosynthesis from the budding yeast *S. cerevisiae*, *S. pombe* and *Candida albicans* were used for BLASTp searches to identify their orthologs in the genome of *F. graminearum*. **(b)** Phenotypic traits of genes involved in protein *O*-glycosylation at the ER (endoplasmic reticulum) and Golgi (golgi apparatus) were examined. virulence, vegetative growth, mycotoxin, and asexual development were categorized as continuous phenotypic traits and scored on a 11-point scale (e: essential, -4: reduced rate between 0 and 0.29, -3: reduced rate between 0.3 and 0.49, -2: reduced rate between 0.5 and 0.69, -1: reduced rate between 0.7 and 0.89, 0: no difference compared with wild-type, + 1: enhanced rate between 1.10 and 0.29, + 2: enhanced rate between 1.30 and 1.49, + 3: enhanced rate between 1.50 and 1.69, +4: enhanced rate between 1.70 and 1.89, +5: enhanced rate over 1.90). Sexual developments were categorized as discrete phenotypic traits and scored on a 4-point scale (a: no difference compared with wild-type, b: decreased number of perithecia and ascospore or delayed perithecia maturation or decreased number of discharged ascospores, c: no perithecia and ascospore formation or no ascospore discharge, e: essential genes). More than three biologically independent experiments were performed for each phenotypic trait. Abbreviations: WH wheat head, CM complete media, MM minimal media, DON deoxynivalenol, ZEA zearalenone, CO conidia, PE perithecia, DI discharge, ACO ascospore.

To comprehensively elucidate the biological functions of protein *N*- and *O*-glycosylation processes in *F. graminearum,* we functionally characterized all resulting gene deletion mutants for defects in virulence, vegetative growth, mycotoxin production, as well as asexual and sexual development. The phenotypic dataset of the mutant collection was organized based on the sequential order of glycan synthesis and visually represented using a color scale (Figs 2, 3, and S3 Table). This systematic phenotypic analysis revealed that approximately 87.6% of the deletion mutants (57 out of 65) exhibited at least one discernible phenotypic change compared to the wild-type strain.

The severity of phenotypic alterations increased notably with the deletion of mutants involved in early glycan biosynthesis. Furthermore, the indispensability of key genes was confirmed, including GDP-mannose pyrophosphorylase (*PSA1*) (*Fg10789*), responsible for synthesizing GDP-mannose from GTP and mannose-1-phosphate; dolichol phosphate mannose (Dol-P-Man) synthase (*DPM*) complex subunits (*Fg24245, Fg09545, Fg14327*), catalyzing the formation of Dol-P-Man from Dol-P and GDP-Man; the oligosaccharyltransferase (*OST, STT*) complex subunits (*Fg20575, Fg01277, Fg23937*), responsible for catalyzing asparagine-linked glycosylation of newly synthesized proteins; and the protein *O*-mannosyltransferase (*PMT*) complex subunits (*Fg03745, Fg01543*), which transfers mannose from dolichyl phosphate-D-mannose to serine and threonine residues of target proteins. These findings underscore the profound impact of glycan structure on various biological functions in *F. graminearum*.

To ascertain correlations among various phenotypes, *in silico N*-/*O*-glycosylation pathway of *F. graminearum*, including the structure of glycan, were constructed, based on the putative glycosylation-related genes (Figs 2a and 3a). Spearman’s rank correlation was computed for glycan mutants exhibiting multiple mutant phenotypes (Fig C in S1 Text). The analysis revealed robust correlations among virulence, vegetative growth, and both asexual and sexual reproduction. Similarly, glycan maturation in the *O*-glycosylation pathway also showed a strong correlation with virulence, vegetative growth, and sexual reproduction, whereas a few genes associated with the *N*-glycosylation pathway exhibited only a moderate correlation. Noteworthy findings include the identification of *OST* (*Fg01117*), *MNS1* (*Fg01555*), *MNL2* (*Fg01661*), and *HOC1* (*Fg06423*) genes, which play distinct roles in virulence independent of vegetative growth. Additionally, DON and ZEA exhibited no significant correlation with both *N*- and *O*-glycosylation pathways. We found that specific truncated glycan structures are closely associated with sexual development, including those: (1) *N*-glycans present before mannosidase-mediated trimming, (2) *N*-glycans lacking sequential extension by Mnn2/5, Mnn14, Mnn4, Mnn1, and Mgat1, and (3) *N*-/*O*-glycans not yet modified by Mnn1 and Mnt2–4. These results indicate that the early phase of glycan mgaturation could affect more diverse phenotypes, including virulence, vegetative growth, and sexual reproduction, and could also have a greater impact on phenotypic severity compared to the late phase of glycan maturation.

As glycan maturation exhibited a discernible correlation with various phenotypic traits except mycotoxin production and asexual development, we analyzed the transcript levels of genes involved in protein glycosylation across these stages (S4 Table and Fig D in S1 Text). The results showed that the genes encoding Ost subunits, which are responsible for transferring Glc_3_Man_9_GlcNAc_2_ to an Asn residue in newly synthesized proteins, were upregulated during vegetative growth and the overall virulence stage. However, these same genes were either downregulated or exhibited no significant difference during sexual and asexual developmental stages.

Additionally, distinct glycosyltransferase genes exhibited stage-specific expression patterns. *ALG5* (*Fg19779*), *MNT2* (*Fg09595*), and *GLS1* (*Fg17395*) were elevated in their transcript levels during vegetative growth, while *ALG13* (*Fg06927*), *ALG14* (*Fg04029*), *ALG12* (*Fg03053*), *MNN10* (*Fg23425*), and *MNN14* (*Fg27773*) were upregulated during sexual developmental stages. In the asexual developmental stage, the transcript level of *MNL2* (*Fg04573*) showed specific elevation at 12 h. Notably, in the virulence stage, *PSA1* (*Fg10789*), *ANP1* (*Fg14905*), and *MNN11* (*Fg15225*) were specifically upregulated. Regarding *O*-glycosylation, *PMT* complex genes, transferring mannose residues from dolichyl phosphate-D-mannose to protein serine/threonine residues at the ER, displayed downregulation during sexual development but upregulation during the virulence stage. These findings imply that distinct or varied glycan structures may be produced at each developmental stage.

### Glycan maturation does correlate with the various stress responses

**Fig 4:**
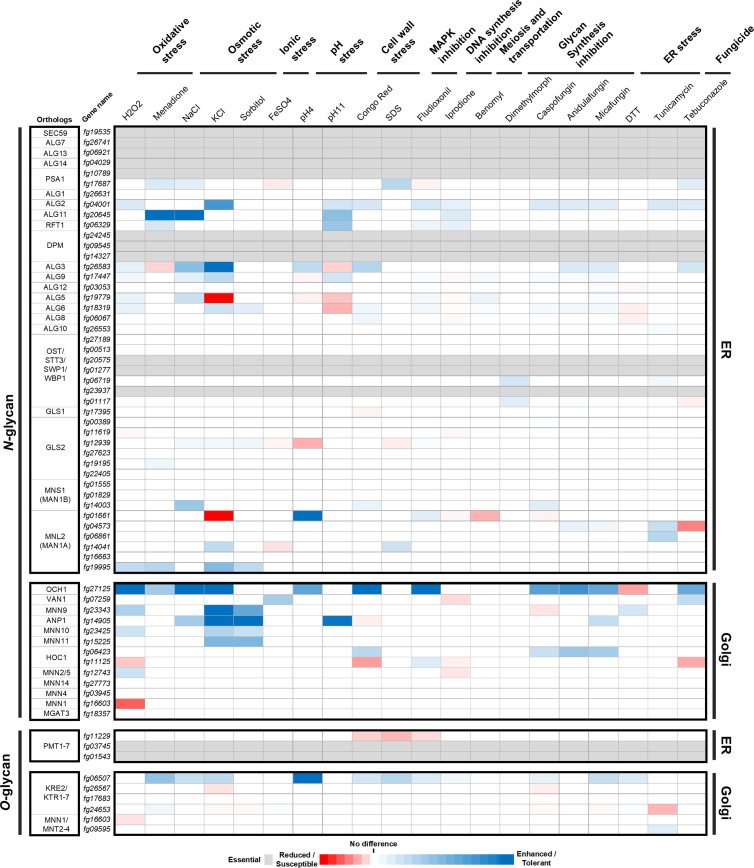
Characterizing in vitro phenotypic traits of genes involved in protein glycosylation. In vitro phenotypic traits were assessed across 20 different growth conditions. The growth score was determined by normalizing the relative growth rates (compared to complete media) to the wild-type and correcting them based on the maximum absolute value observed under each stress condition. The color code is as follows: the light grey indicates essential genes, red indicates reduced or susceptible genes, and blue indicates enhanced or tolerant genes. Each phenotypic trait was evaluated in more than three biologically independent experiments. Abbreviations: H2O2 CM + 7 mM H2O2, Menadione CM + 38 μM Menadione, NaCl CM + 1 M NaCl, KCl CM + 1 M KCl, Sorbitol KCl CM + 1.5 M Sorbitol, FeSO4 CM + 5 mM FeSO4, SDS CM + 100 mg/L sodium dodecyl sulfate, Congo Red CM + 400 mg/L Congo Red, Fludioxonil CM + 0.023 mg/L Fludioxonil, Iprodione CM + 8.6 mg/L Iprodione, Benomyl CM + 0.1 mg/L Benomyl, Dimethomorph CM + 2.5 μg/ml Dimethomorph, Caspofungin CM + 0.2 μg/ml Caspofungin, Anidulafungin CM + 0.03 μg/ml Anidulafungin, Micafungin CM + 0.02 μg/ml Micafungin, DTT CM + 10 mM dithiothreitol, Tunicamycin CM + 2.5 μg/ml Tunicamycin, Tebuconazole CM + 0.1 mg/L Tebuconazole.

We examined the responses of deletion mutants under 20 distinct *in vitro* stress conditions, including oxidative stress, osmotic stress, ionic stress, pH stress, cell wall stress, MAPK inhibition condition, DNA synthesis inhibition condition, meiosis and transport inhibition condition, cell wall glycan synthesis inhibition condition, ER stress, and several fungicide treatment conditions (Figs 4 and E in S1 Text, and S5 Table). The stress response dataset of mutants involved in protein glycosylation was quantitatively represented using a color scale. Apart from essential genes, our analysis revealed that approximately 76.9% of the deletion mutants (40/52) exhibited at least one discernible phenotypic defect. Interestingly, the majority of stress responses demonstrated resistance (72.8%) rather than susceptibility (27.2%). Nevertheless, we did not observe any significant correlation between glycan maturation and stress responses (Fig F in S1 Text).

### The composition of cell wall components and glycan structures showed notable differences between conidia and mycelia

A significant enrichment of glycoproteins was observed in the cell membrane and extracellular regions, including the cell wall [66,67]. In a recent study, we highlighted the distinct regulation of cell wall proteins across developmental stages [57]. Given the variability in gene expression patterns of the protein glycosylation pathway across developmental stages (S4 Table and Fig D in S1 Text), we explored whether the glycan structures of cell wall glycoproteins also exhibited variations across these stages. Due to the intricate internal structures of perithecia, representing the sexual reproduction stage, sampling from this stage was not feasible. Furthermore, samples were not collected from *F. graminearum*-infected wheat tissue, representing the virulent stage, due to challenges in separating invasive hyphae from host tissue. Consequently, our investigation focused on mycelia and conidia, representing vegetative growth and asexual development, respectively.

**Fig 5:**
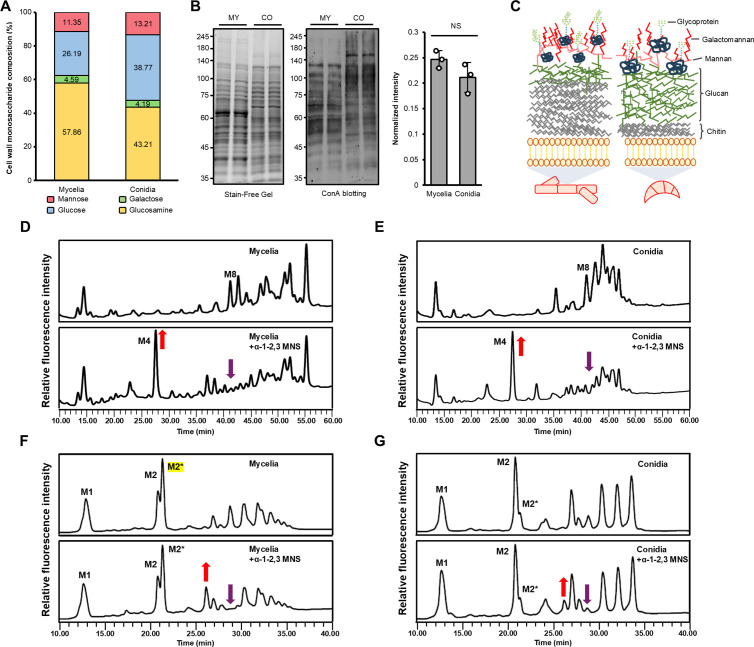
The proportion of cell wall components and glycan structures differed between conidia and mycelia. **(a)** Monosaccharides composition of cell walls between conidia and mycelia. Fucose and galactosamine were not detected. **(b)** Different glycoprotein levels in mycelia and conidia were assessed by western blot using the Con A-HRP antibody. MY, mycelia; CO, conidia. The intensity of each lane, as observed after lectin blotting, was normalized by the intensity of the respective sample from the stain-free gel. **(c)** Schematic diagram illustrating the differences in cell wall components, glycoproteins, and glycan structures between mycelia and conidia. **(d-e)** HPLC profiles of *N*-glycans of cell wall mannoproteins (cwMPs) from the mycelia and conidia of the wild-type strain Z-3639 of *F. graminearum*. Normal-phase HPLC was conducted using a Shodex Asahipak NH2P-50 4E column (Showa Denko) (0.46 by 25 cm) with solvent A (2% glacial acetic acid–1% tetrahydrofuran–acetonitrile) and solvent B (5% glacial acetic acid–3% triethylamine–1% tetrahydrofuran–HPLC water) at a flow rate of 1.0 ml/min. After sample injection, the proportion of solvent B was maintained at 30% for 5 min and then increased linearly to 70% over 80 min. + *α*-1-2,3 MNS, *N*-glycans treated with *α*-1-2,3 MNS. **(f-g)** *O*-glycans of cwMPs from the mycelia and conidia of the wild-type strain Z-3639 of *F. graminearum*. Normal-phase HPLC was conducted using a TSKgel Amide-80 column (Tosoh Bioscience) (0.46 by 25 cm) with solvent A (2% glacial acetic acid–1% tetrahydrofuran–acetonitrile) and solvent B (5% glacial acetic acid–3% triethylamine–1% tetrahydrofuran–HPLC water) at a flow rate of 1.0 ml/min. After sample injection, the proportion of solvent B was maintained at 10% for 5 min and then increased linearly to 90% over 60 min. + *α*-1-2,3 MNS, *O*-glycans of treated with *α*-1-2,3 MNS.

To elucidate the monosaccharide composition of glycan structures, we conducted an analysis of cell wall components (Fig 5a and S6 Table). This analysis unveiled higher proportions of glucosamine, the chitin precursor, in mycelia compared to conidia. Conversely, the proportion of glucose, the glucan precursor, was higher in conidia than in mycelia. No significant differences were observed in the proportions of mannose and galactose between mycelia and conidia, and fucose and galactosamine were not detected. We additionally analyzed the monosaccharide composition of cell wall glycoproteins to determine whether galactose is present in the cell wall itself or in the glycan structures attached to cell wall glycoproteins. This analysis revealed that galactose was not detected in the cell wall glycoproteins, implying that it was only present in the cell wall (Fig 5c and S6 Table).

We further conducted a comparative analysis of glycosylation levels in the two different stages. Lectin blotting using concanavalin A (Con A) revealed no difference in the ratio of cell wall glycoprotein (Fig 5b). However, more glycoproteins with high molecular weight were detected in conidia than in mycelia. These results highlight not only the differences in the proportion of chitin and glucan matrix between mycelia and conidia but also the higher abundance of glycoproteins with high molecular weight in conidia compared to mycelia (Fig 5c).

Based on the analysis of cell wall components, we anticipate that the glycan structure of cell wall glycoproteins would mainly consist of mannose, galactose, glucose, and glucosamine. To gain insight into the general structure of *N*- and *O*-glycans in *F. graminearum*, we conducted high-performance liquid chromatography (HPLC)-based glycan analysis on both mycelia and conidia. The glycans assembled on cell wall mannoproteins (cwMPs) from the wild-type (WT) strain Z-3639 of *F. graminearum* were obtained, labeled with 2-aminobenzoic acid, and analyzed by Normal-phase HPLC. The results revealed distinctive *N*-glycan profiles between mycelia and conidia, ranging from approximately 8–20 monosaccharides in size. In mycelial profile, the peaks corresponding to the *N*-glycans containing eight (M8, GlcNAc_2_Man_8_) or more mannose residues were distributed from 40 min to 55 min range, whereas in conidia *N*-glycan profile, the peaks larger than M8 were predominantly detected within the 40–50 min range. The treatment with *α*-1,2- and *α*-1,3-mannosidase (MNS), specifically cleaving *α*-1,2- and *α*-1,3-mannose linkages, led to an overall reduction in glycans observed between 40 and 48 min, converting most glycan peaks to a smaller peak corresponding to four mannose residues (M4) in both mycelia and conidia. In contrast, the peaks observed after 51 min in the mycelia were not significantly affected by the enzyme treatment (Fig 5d-e). The results indicated that the *N*-glycans of *F. graminearum* observed between 40 and 48 min are mostly composed of mannose residues in *α*-1–2 and 1–3 linkages. However, the remaining peaks suggest the presence of another mannose linkage, such as *α*-1, 6, or the addition of other monosaccharide besides mannose.

The *O*-glycan profiles of *F. graminearum* revealed that *O*-glycans are relatively small, typically consisting of one to six monosaccharide residues (Fig 5f-g). Two distinct peaks were detected as the glycan peak composed of two mannose residues (M2 and M2*), suggesting the presence of two isomer forms in different linkages. As observed in the *N*-glycan profiles, the overall *O*-glycan peak patterns of mycelia and conidia displayed a few noticeable differences. Besides the differential proportion of M2 isomers, mycelia appear to contain less extended *O*-glycans (M3 ~ M6) compared to conidia. In contrast to *N*-glycans, the *O*-glycan peaks exhibited only partial alterations upon *α*-1–2, -3 MNS treatment, indicating that the terminal mannose linkages of *F. graminearum O*-glycan might be *α*-1,6 rather than *α*-1,2 or *α*-1,3, or suggesting that other monosaccharide besides mannose might be present as terminal sugar.

Next, we investigated whether the changes in glycan abundance resulted from alterations in the transcript levels of corresponding biosynthetic enzymes. Notably, analysis of previously published transcriptome data revealed higher expression levels of most *N*-mannosyltransferase-encoding genes at the mycelial stage compared to conidia (Fig D in S1 Text), consistent with the more abundant presence of hypermannosylated -type of *N*-glycans in mycelia (Fig 5d-e). Also, the expression levels of most *O*-mannosyltransferase-encoding genes were downregulated in mycelia compared to conidia, consistent with the *O*-glycna profiles exhibiting more extended *O*-glycans in conidia. This correlation between the transcriptome data with glycan profile data strongly indicated that the abundance of glycans with different structures is mainly determined by the expression levels of glycan processing genes at transcription.

### *ALG3* (*Fg26583*) and *ALG12* (*Fg03053*) are implicated in various developmental processes and *N*-glycan biosynthesis

**Fig 6:**
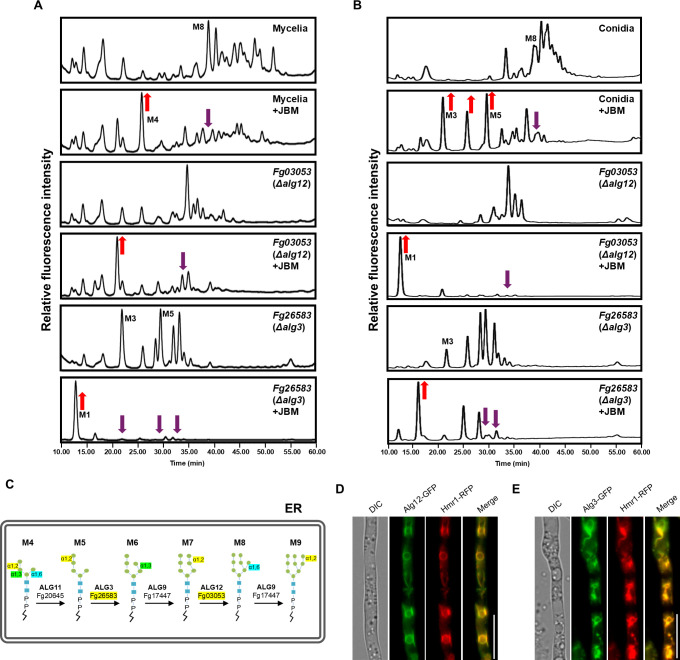
*F. graminearum* Fg03053 (Alg12) and Fg26583 (Alg3) are involved in *N*-glycan biosynthesis within the ER lumen. (a) HPLC profiles of *N*-glycans of cell wall mannoproteins (cwMPs) from the mycelia and conidia of the wild-type, Δ*fg03053,* and Δ*fg26583*. Normal-phase HPLC was conducted using a Shodex Asahipak NH2P-50 4E column (Showa Denko). + JBM, *N*-glycans treated with *α*- jack bean *α*-1,2,3,6 MNS. (b) Partial schematic diagram illustrating the difference in final glycan structures between the wild-type and each mutant strain Δ*fg03053,* and Δ*fg26583*. (c) Partial schematic diagram illustrating the difference in final *N*-glycan structures between the wild-type and each mutant strain Δ*fg03053,* and Δ*fg26583*. (d) Subcellular localization of Fg03053 (Alg12) and Fg26583 (Alg3). Fg03053 and Fg26583 were fused with green fluorescent protein (GFP) and Hmr1, an endoplasmic reticulum (ER) marker, was tagged with red fluorescent protein (RFP). The yellow color in the merged images indicates co-localization. Scale bar = 10 μm.

While most glycosyltransferases involved in the initial *N*-glycosylation at the ER are encoded by a single gene for each glycan synthesis reaction, the late *N*-glycosylation at the Golgi and *O*-glycosylation involve multiple glycosyltransferases. This implies that these complex subunit genes are genetically redundant. Based on a previous study in *C. neoformans*, where deletion of *ALG3*, *ALG9*, or *ALG12*—encoding dolichyl-phosphate-mannose (Dol-P-Man)-dependent mannosyltransferases involved in ER luminal lipid-linked *N*-glycan biosynthesis—led to the accumulation of truncated core-*N*-glycans [15], we aimed to investigate the roles of the corresponding genes in *F. graminearum*. As shown in Figs 2a and 6c, Alg3 (Fg26583), Alg9 (Fg17447), and Alg12 (Fg03053) function sequentially in the *N*-glycan biosynthesis pathway: Alg3 initiates the addition of an *α*-1,3-mannose to Dol-PP-GlcNAc₂Man₅, followed by the addition of *α*-1,2- and *α*-1,6-mannoses by Alg9 and Alg12, respectively. Notably, Alg9 has a dual role—adding both the initial *α*-1,2-mannose and capping the *α*-1,6-mannose branch with another *α*-1,2-mannose—placing it both upstream and downstream in the pathway. Deletion of *ALG3* and *ALG12*, but not *ALG9*, led to significant phenotypic defects. This is likely due to the critical positioning of their enzymatic steps in core *N*-glycan assembly: Alg3 acts early to initiate *α*-1,3 mannose branching, while Alg12 performs the penultimate *α*-1,6-mannose addition required for completing the core structure. In contrast, the terminal *α*-1,2-mannose addition catalyzed by Alg9 may be partially dispensable or compensated by redundant mechanisms via additional α-1,2-mannosyltransferases in the Golgi such as *MNN2/5*, resulting in milder phenotypes upon deletion. Therefore, our functional analysis was focused on the genes involved in the biosynthesis of core *N*-glycans in the ER, particularly by examining the roles of *ALG3* (*Fg26583*) and *ALG12* (*Fg03053*), as their deletion resulted in various phenotypic defects including virulence (Fig 2 and S3 Table).

Alg12 (Fg03053) encodes 552 amino acids and contains the Dol-P-Man:Man_7_GlcNAc_2_-PP-Dol *α*-1,6-mannosyltransferase domain (IPR039485), while Alg3 (Fg26583) encodes 432 amino acids and contains the glycosyltransferase, Alg3 domain (IPR007873). Both proteins contain 11 and 5 transmembrane domains, respectively, and are predicted to be localized in the ER. We further examined the degree of sequence and domain conservation between *F. graminearum* and the other fungal species (S3 Table). Although the protein sequences of yeast-form fungi exhibited lower conservation compared to filamentous fungi, the sequence identities in all species and their functional domains were retained throughout evolution. Given this level of sequence conservation, we assume that Alg12 (Fg03053) and Alg3 (Fg26583) function as *α*-1,6-mannosyltransferase and Dolichol-P-Man-dependent *α*-1,3-mannosyltransferase, respectively.

HPLC profiles were conducted on *N*-glycans obtained from cwMPs of mycelia and conidia samples for each mutant strain and compared to that of the WT glycans (Fig 6a, b). Compared to the WT glycans, the *N*-linked oligosaccharides, specifically the M8 and hypermannosylated peaks (larger than M8), were markedly reduced in both mutants. Conversely, the M3 to M6 peaks (GlcNAc_2_Man_3–5_) and M6-M7 peaks (GlcNAc_2_Man_6–7_) were increased in the *ALG3* (*Fg26583*) and *ALG12* (*Fg03053*) deletion mutant strains, respectively, compared to that of the WT strain. The overall *N*-glycan profiles of mycelia and conidia became quite similar upon deletion of *ALG3* (*Fg26583*) and *ALG12* (*Fg03053*), due to the almost absence of outer chain biosynthesis causing differential degree of hypermannosylation. Treatment with jack bean MNS, cleaving *α*-1,2,3,6-linked mannose residues, led to the shift of the *N*-glycan peaks to M1 (GlcNAc_2_Man_1_) in *AGL3* deletion or to M1 ~ M3 in the *AGL11* deletion mutant strain. These results suggest that *ALG3* (*fg26583*) deletion strain is defective in the conversion of Dol-PP-GlcNAc_2_Man_5_ to Dol-PP-GlcNAc_2_Man_6_, which constitutes the initial step in attaching a mannose residue to the lipid-linked *N*-oligosaccharide in the ER lumen, and *ALG12* (*fg03053*) deletion strain is defective in the conversion of Dol-PP-GlcNAc_2_Man_7_ to Dol-PP-GlcNAc_2_Man_8_ (Fig 6c).

Because glycans core *N*-glycans are synthesized in the ER, we generated transgenic strains expressing Alg12 or Alg3 fused with green fluorescent protein (GFP) under the control of a constitutive promoter RP27 [68], alongside the ER marker FgHmr1 fused with red fluorescent protein (RFP) [69]. Both Alg12-GFP and Alg3-GFP were co-localized with the Hmr1-RFP, indicating that the subcellular localization of Alg12 and Alg3 is in the ER, consistent with their orthologs in *S. cerevisiae* (Fig 6c)*.*

### Deletion of *ALG3* (*Fg26583*) and *ALG12* (*Fg03053*) affect the glycosylation pattern of Cnb1, Gic1, and Glx, which are essential for the virulence of *F. graminearum*

**Fig 7:**
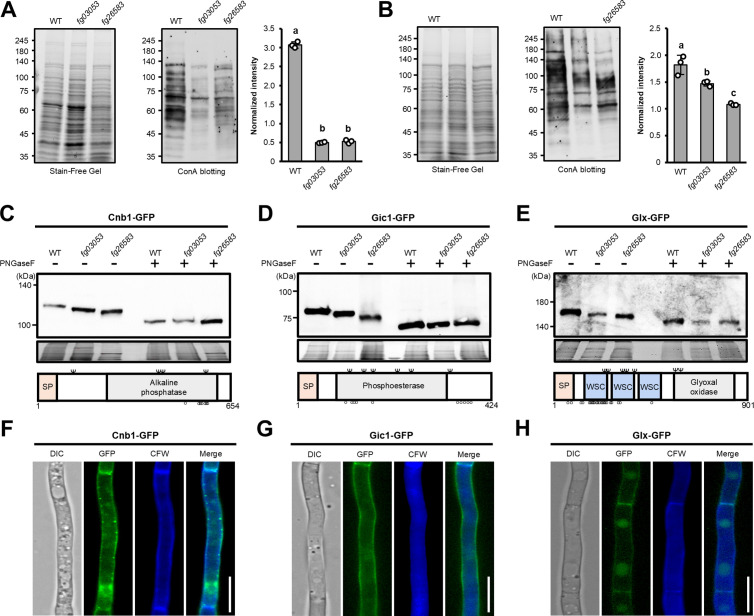
Fg03053 and Fg26583 are involved in the *N*-glycosylation of glycoproteins Cnb1, Gic1, and Glx, which are required for the virulence of *F. graminearum.* **(a,b)** Lectin blotting analysis of mycelia and conidia stages. Gycoprotein levels of the wild-type, Δ*fg03053,* and Δ*fg26583* were assessed by western blot using the Con A-HRP antibody. The intensity of each lane in the lectin blot was normalized by the intensity of the respective sample from the stain-free gel. Mean and standard deviation of the intensity of each strain were estimated with data from three (*n* = *3*) independent biological replicates (marked with white dots on the bars). Different letters indicate significant differences based on analysis of variance (ANOVA) followed by Bonferroni test (*P* = 0.01). **(c-e)** Western blot analysis using an anti-GFP antibody for detection of under-glycosylated cell wall proteins Cnb1, Gic1, and Glx in Δ*fg03053,* and Δ*fg26583.* SP, signal peptide domain; WSC, cell wall integrity and stress response component domain; branch symbol, putative *N*-glycosylation site; circle, putative *O*-glycosylation site. *N*- or *O*-glycosylation sites were predicted using NetNGlyc 1.0 and NetOGlyc 4.0. (f-h) Subcellular localization of Cnb1, Gic1, and Glx. Mycelia were grown in liquid complete media for 24 **h.** Calcofluor white (CFW) staining visualizes cell walls and septa. GFP and CFW fluorescence images were superimposed in the ‘Merge’ panel. Scale bar = 10 μm.

*N*-glycosylation plays pivotal roles in glycoprotein modification and the ER-mediated protein quality control (ERQC) system [24,26]. Lectin blotting using Con A revealed that the total intensities of bands detecting glycoproteins of *alg3* (*fg26583*) and *alg12* (*fg03053*) were reduced by more than 2-fold compared to the wild type in both mycelia and conidia (Fig 7a and b). This finding suggests that the loss of *ALG3* (*Fg26583*) and *ALG12* (*Fg03053*) are not only involved in glycan synthesis but also in modulating the total amount of glycoprotein. This might be probably due to decreased stability of glycoproteins with truncated glycans or inefficient transfer of truncated core-*N*-glycans to proteins via Ost, resulting overall reduction of glycosylation.

To investigate whether *ALG3* (*Fg26583*) and *ALG12* (*Fg03053*) are involved in the glycosylation of their target glycoproteins, we focused on the glycosylation patterns of three glycoproteins identified from our previous cell wall glycoproteome data [57], known for their involvement in the virulence of *F. graminearum*: glyoxal oxidase (Glx), which catalyzes hydrogen peroxide production [70], and the two phosphatases (Cnb1 and Gic1) [38,70]. Cnb1, Gic1, and Glx were predicted to harbor 4, 6, and 7 glycosylation sites, respectively, and signal peptides at the N terminus. Immunoblot analysis revealed that the GFP-tagged Cnb1, Gic1, and Glx in *alg3* (*fg26583*) and *alg12* (*fg03053*) mutant strains displayed lower molecular weights compared to those in the wild-type strain (Fig 7c-e). Upon PNGase F treatment, which cleaves *N*-linked oligosaccharides from glycoproteins, the protein sizes converged to the same molecular weight in both mutant strains and the wild-type strain. This indicates that truncated *N*-glycans with reduced size were attached to Cnb1, Gic1, and Glx proteins in the *alg3* (*fg26583*) and *alg12* (*fg03053*) mutant strains.

Additionally, we confirmed the subcellular localization of Cnb1, Gic1, and Glx under a fluorescence microscope with Calcofluor White staining, showing that all three proteins were primarily localized in the cell wall (Fig 7f-h). This finding suggests that Cnb1, Gic1, and Glx are cell wall glycoproteins modified with the attachment of *N*-glycans, which are synthesized by Alg3 (Fg26583) and Alg12 (Fg03053)-mediated early *N*-glycosylation pathway in *F. graminearum*.

### Quantitative glycoproteomics profiling and functional classification of identified glycoproteins in *F. graminearum*

To elucidate downstream target glycoproteins, whose stability might be affected by truncation of *N*-glycans due to the loss of Alg3 (Fg26583) and Alg12 (Fg03053), we employed a label-free quantitative glycoproteomic approach [71]. To determine whether the difference in peptide counts between the WT strain and the mutants originated from the glycoprotein degradation in the deletion mutants or arose from altered glycoprotein binding affinity to Con A due to abnormal glycan formation in the deletion mutants, we utilized the global proteome as a control and loaded an equal amount of protein for each sample analysis (Fig G in S1 Text). If a protein was enriched more than two-fold in the Con A-eluted sample compared to the global proteome, we defined it as a glycoprotein. We also included proteins orthologous to or overlapping with the *N*-glycoproteome of *G. graminicola, M. oryzae,* or *F. graminearum* in previous studies [23,24,26]. Following detection and identification via liquid chromatography–tandem mass spectrometry (LC-MS/MS) analysis, we identified 406 glycoproteins among 1159 total proteins, representing 8.2% of the total proteins in *F. graminearum*, with high confidence (S7 Table).

The equal spectral counts of the housekeeping protein glyceraldehyde 3-phosphate dehydrogenase (GAPDH) indicate that all protein samples were analyzed in equal amounts. Principal component analysis (PCA) revealed that the global proteome and glycoproteome from each strain differed in spectral counts patterns, implying that glycoproteome was well-extracted in this experiment (Fig H in S1 Text). Of the 406 glycoproteins identified, 274 had been previously identified in prior *N*-glycoproteomic studies (S7 Table). These results not only support the efficient extraction of *N-*glycoproteins but also suggest that the remaining 132 proteins could be considered to have only *O*-glycans.

To predict the role of glycoproteins in *F. graminearum,* we conducted Gene Ontology (GO) functional classification and Kyoto Encyclopedia of Genes and Genomes (KEGG) pathway analyses. We found that most glycoproteins were localized to extracellular regions, cell wall, plasma membrane and ER. (Fig I in S1 Text). These results align with the understanding that glycoproteins primarily function in the ER, membrane-bounded organelles, and secretory system. The largest proportion of glycoproteins were involved in cell wall organization or biogenesis, carbohydrate metabolism, protein folding and proteolysis. For the molecular functions, the majority of glycoproteins were involved in protein folding and peptidases, supporting the key roles of protein glycosylation associated with protein quality control and amino acid cycling. Proteins with predicted structural molecule activity and pyrophosphatase activity were also glycosylated

KEGG pathway enrichment analysis revealed that glycoproteins are most enriched in the biosynthesis of secondary metabolites, protein processing in the ER, and carbon metabolisms including glycolysis/gluconeogenesis and starch and sucrose metabolisms. Protein processing in the ER, which is closely associated with *N*-glycosylation as shown in previous studies, plays a crucial role in protein quality control and transport. We identified 17 glycoproteins involved in protein processing in the ER, including Nef and Bip for protein recognition by luminal chaperones; Ost, Gls1, Gls2, Cnx, Uggt, Ergic53, Ero1, Os9, and Pdis for protein quality control in the ER; and Hsp70, Hsp40, Skp1, and P97 for ER-associated protein degradation. Thus, protein glycosylation might play essential roles in protein processing and quality control in the ER by affecting the function, stability, or localization of proteins involved in the ERQC pathway.

Taken together, these results suggest that the glycoproteins of *F. graminearum* are linked to several important biological processes including protein quality control, amino acid cycling, and carbon metabolism, which are considered key components of pathobiological processes and are primarily localized at the cell wall and extracellular regions.

### Glycosyltransferases involved in the early core *N*-glycosylation pathway influence global protein glycosylation across various biological processes

Pearson correlation analysis revealed no significant correlation between the global proteome and glycoproteome profiles, indicating that differential spectral counts observed in the *∆alg3* and *∆alg12* mutants were not due to general changes in protein expression. Instead, they likely reflect impaired glycoprotein enrichment efficiency, resulting from defective glycan structures and altered binding affinity to Con A (Fig J in S1 Text).

Comparing the spectral counts of Con A-eluted samples between wild-type and mutant strains, we identified 295 (72.6%) and 249 (61.3%) differentially glycosylated proteins in alg3 (*fg26583*) and *alg12* (*fg03053*) mutants, respectively*.* Absence of Alg3 (Fg26583) was found to affect more glycoproteins than Alg12 (Fg03053), consistent with the Con A-blotting results showing fewer glycoproteins in the *alg3* (*fg26583*) sample.

**Fig 8:**
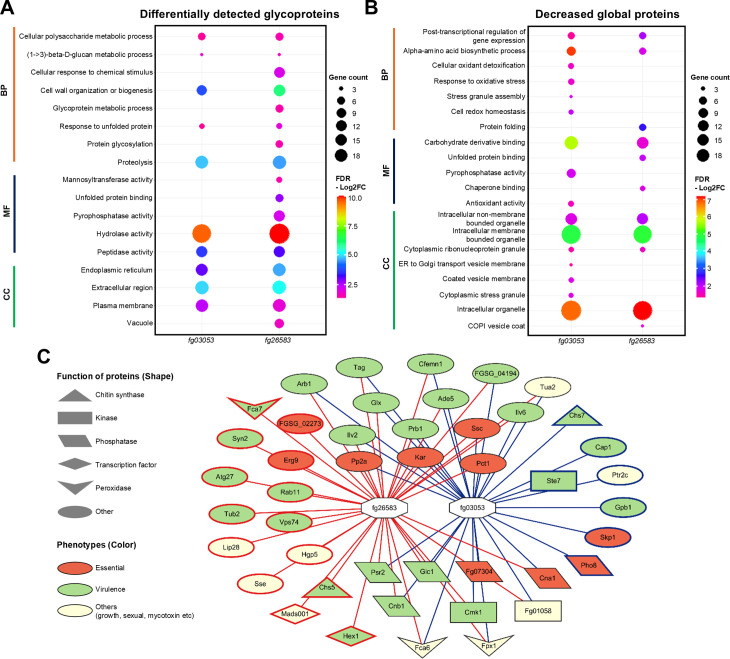
Glycoproteomic analysis of wild type. **Δ*fg03053 (alg12),* and** **Δ*fg26583 (alg3).*** The dot plot illustrates enriched Gene Ontology (GO) terms related to biological processes for (a) identified glycoproteins and (b) downregulated proteins in the total proteome of the wild type, Δ*fg03053*, and Δ*fg26583* strains. The size of the dot represents the percentage of the gene count, and the color indicates the FDR, false discovery rates. **(c)** Protein-protein interactions (PPIs) of glycoproteins associated with phenotypic defects affected by Fg03053 and Fg26583. 43 glycoproteins were visualized by the Cystoscope program. The functions of the proteins were categorized by shape, and the phenotypes were represented using different colors. Additionally, proteins that might be affected solely by Fg03053 or Fg26583 were marked with blue or red borders, respectively.

Gene Ontology annotation of all identified glycoproteins from total global proteomics revealed their potential roles in biological processes, molecular functions, and cellular components (Fig 8a). Most glycoproteins were predicted to localize to the ER, membrane, and extracellular regions. Notably, glycoproteins localized to the vacuole were exclusively identified in *alg3* (*fg26583*). These glycoproteins were linked to polysaccharide metabolic processes, cell wall organization, and protein quality control (e.g., response to unfolded protein and proteolysis) in both *alg3* (*fg26583*) and *alg12* (*fg03053*). Additionally, proteins unique to *alg3* (*fg26583*) were involved in glycosylation (protein glycosylation and glycoprotein metabolic processes) and the cellular response to chemical stimuli. In molecular function classification, most glycosylated proteins were predicted to have hydrolase activity in both *alg3* (*fg26583*) and *alg12* (*fg03053*). Additionally, glycoproteins with predicted mannosyltransferase activity, unfolded protein binding, and pyrophosphatase activity were exclusively identified in *alg3* (*fg26583*).

We also identified 193 (16.7%) and 223 (19.2%) proteins that were downregulated in the *alg3* (*fg26583*) and *alg12* (*fg03053*) mutants, respectively*.* In contrast to glycoproteins, GO functional classification analysis revealed that most global proteins were localized to intracellular organelles and the cytoplasm (Fig 8b). Regarding biological processes and molecular functions, proteins associated with oxidative stress were predominantly downregulated in alg12 (fg03053), while those involved in protein quality control were primarily downregulated in alg3 (fg26583). Collectively, our proteomic analysis results highlight that the structure of core N-glycans assembled in the ER is crucial for modulating the steady-state levels of glycoproteins involved in various biological processes, primarily localized to extracellular regions, as well as proteins associated with diverse intracellular processes.

### Identification of virulence-related glycoproteins affected by early core *N*-glycosylation in *F. graminearum*

Among the glycoproteins affected by the loss of Alg3 (Fg26583) or Alg12 (Fg03053), we identified 60 glycoproteins previously characterized in functional studies [36–38,40,44,70, 72–99]. Of these, 24 were associated with virulence, 10 were essential, and 9 were linked to phenotypes not directly related to virulence, including vegetative growth, sexual development, mycotoxin production, and stress sensitivity (Fig 8c and S7 Table). These glycoproteins participate in diverse cellular processes and include transcription factors (Mads001 and Hex1), phosphatases (Cna1, Cnb1, Pp2a, Gic1, Ptc5, Pho8, Psr2, Fg09475, Fg03986, and Fg07304), kinases (Ste7 and Cmk1), peroxidases (Fpx1, Fca6, and Fca7), 70-kDa heat shock proteins (Kar, Ssc, and Sse), lipases (Lip28 and Tag), chitin synthases (Chs5 and Chs7), proteins involved in autophagy (Rab11, Vps74, Atg27), or microtubule function (Tua2, Tub2 and Cap1) (S7 Table).

A protein-protein interaction network analysis revealed that 23 glycoproteins were co-regulated by Alg3 (Fg26583) and Alg12 (Fg03053), while 14 were specifically regulated by Alg3 (Fg26583), and 7 were exclusively regulated by Alg12 (Fg03053). Altogether, these findings suggest that the functional defects of Alg3 (Fg26583) and Alg12 (Fg03053), key players in the early core *N*-glycosylation pathway, affect a wide range of glycoproteins by generating altered core *N*-glycan structures, modulating the amount and function of glycoprotein and ultimately impacting virulence in *F. graminearum*.

## Discussion

Among the various post-translational modifications that enhance the functional diversity of the proteome, protein glycosylation is one of the most common, influencing protein folding, stability, quality control, transport, and localization. Recent studies have investigated several genes involved in protein glycosylation across various plant pathogenic fungi, such as *M. oryzae*, *G, graminicola*, and *B. cinerea*, revealing their crucial roles in diverse biological processes, including pathogenicity and vegetative growth [20,21,24,26]. In particular, protein glycosylation has been shown to play an important role in effector secretion in *M. oryzae* and *G, graminicola* [20,26]. However, most of these studies have focused on individual genes, and comprehensive functional genomic analyses of the glycosylation pathway—as well as detailed characterization of glycan structures and glycoproteomes—remain limited in plant pathogenic fungi. Protein glycosylation has been shown to play critical roles in virulence, vegetative growth, sexual and asexual reproduction processes in fungi [17,24,26]. However, comprehensive glycomics study exploring how protein glycosylation modulates the pathogenicity of plant pathogenic fungi is still largely unknown. In this study, (i) we present the first large-scale functional genomics analysis of 65 glycosylation-related genes in *F. graminearum*, providing a systems-level understanding of the glycosylation pathway. (ii) We also uncovered stage-specific differences in *N*- and *O*-glycan structures, highlighting a previously unrecognized layer of glycan-mediated developmental regulation. (iii) Furthermore, we identified 34 glycoproteins previously characterized as essential or virulence-associated, suggesting that these targets could serve as promising candidates for the development of broad-spectrum antifungal agents.

Comparative analysis showed that mannosidases were markedly more abundant in filamentous fungi than in yeast-form fungi (Fig 1). *α*-mannosidases serve diverse cellular functions and are localized in various compartments, playing key roles in *N*-glycan processing within the endoplasmic reticulum and Golgi apparatus. Phylogenetic analysis based on protein sequence alignments divides *α*-mannosidases into two evolutionary distinct classes, known as Class I and Class II [100]. In higher eukaryotes, Class I *α*-1,2-mannosidases trim up to four mannose residues, producing Man5GlcNAc2, the precursor for complex *N*-glycans in the Golgi. These glycans are further processed through addition of GlcNAc by *N*-acetylglucosaminyl transferase, sequential removal of two mannose residues are removed by *α*-mannosidase II, and additional sugars such as galactose and sialic acid are incorporated [101,102]. However, the lower eukaryote *S. cerevisiae* possesses a single ER-specific *α*-1,2-mannosidase, which cleaves one mannose residue from the core oligosaccharide, yielding Man8GlcNAc2 [103], without the Golgi-associated mannosidases. In terms of diversity, the *α*-mannosidase gene family in filamentous fungi appears to be more comparable to mammalian systems than to yeast. The two kinds of *α*-1,2-mannosidases were identified in *A. oryzae* and *Aspergillus saitoi* [104,105]. In the ER, the first *α*-1,2-mannosidase cleaves all four *α*-1,2-linked mannose residues from Man₉GlcNAc₂ to generate Man₅GlcNAc₂ and has been identified in *A. oryzae* and *A. saitoi*. In *A. oryzae*, another *α*-1,2-mannosidase has been identified that cleaves a single mannose residue from the core oligosaccharide in the Golgi, forming Man₈GlcNAc₂ [106]. Three different *α*-mannosidase genes have been also identified in *A. nidulans* [107]. Our phenotypic data also confirms that multiple mannosidases in *F. graminearum* function redundantly (Fig 2). This suggests that mannose removal during *N*-glycan processing in filamentous fungi could involve multiple *α*-mannosidases with overlapping functions, potentially minimizing the physiological effects of individual enzyme disruptions. Such redundancies have also been observed in other lineages, including *Arabidopsis* [108], Human [109], and mice [110]. Future studies should further investigate the localization and function of fungi-specific multiple mannosidases in glycan structure processing.

We also found that 23 genes were identified as essential in at least one species. Among these, ten genes exhibited lethality exclusively in *S. cerevisiae* and *S. pombe*, but were non-essential in *F. graminearum*. Of these ten genes, only three were found to have paralogous copies in filamentous fungi.

Similar to the diversity observed in glycosyltransferases across species, glycan structures also exhibit species-specific differences. Glycan recognition can be broadly categorized into two classes: endogenous recognition by receptors within the same organism and exogenous recognition by other organisms, such as pathogenic or symbiotic microbes [103]. Most pathogenic fungi must first attach to host cells via recognition of specific glycan structures, suggesting that these fungi-host interactions based on glycan recognition are subject to distinct evolutionary selection pressures. For instance, in *Aspergillus* species, sialic acids—negatively charged carbohydrates present on the conidial surface—are found at higher densities in pathogenic strains compared to non-pathogenic ones, suggesting a potential role in virulence [111]. Additionally, a previous study concluded that diverse mannosidase with different linkage types and lengths cannot be considered as strict virulence factors, as they are also present in the cell walls of non-pathogenic yeasts [112]. Therefore, it is plausible that specific monosaccharides, such as galactose or sialic acids, which are uniquely enriched in certain species, may be associated with host-related adaptations. Although some efforts have been made to investigate the evolution of glycosyltransferases and glycan structures in relation to host factors across pathogenic and non-pathogenic fungi, our understanding of glycan diversity and its host-associated evolution remains limited—particularly in comparative studies among animal pathogens, plant pathogens, and non-pathogenic fungi. Therefore, further comparative evolutionary studies are needed to clarify whether glycosylation-related genes display distinct distribution patterns among fungal species with different host specificities, and to what extent their evolution is driven by host-associated selective pressures.

Our present study strongly indicated that glycan maturation in early phase affects a broader range of phenotypes exerting a greater impact on phenotypic severity, compared to the glycan maturation in the late phase. Previous studies have demonstrated that both *N*-glycan structures and *N*-glycosylation sites vary across developmental stage. In *Oryza sativa*, for instance, shoots and roots exhibit distinct glycoprotein profiles and *N*-glycan biosynthetic pathways [113]. Similarly, in *M. oryzae*, *N*-glycosylation has been shown to coordinate specific cellular processes required for hyphal growth, conidiation, and appressorium formation [24]. Our data on the comparison of glycan structures between mycelia and conidia of *F. graminearum* also revealed the distinctive glycan profiles in both *N*- and *O*-glycans (Fig 5). Transcriptome data showed significant correlation between the expression and functional patterns of glycosylation-related proteins, suggesting that the abundance of structurally distinct glycans is largely governed by the transcriptional regulation of glycan-processing genes in *F. graminearum*. These findings imply that stage-specific glycosylation phenotypes may result not only from differences in the glycosylation machinery, but also from variation in the expression and function of glycosylated proteins required at each developmental stage. Further studies would be needed to identify the proteins glycosylated differentially at different stages, which might modulate their functions specific to distinct developmental phenotypes.

The fungal cell wall is crucial not only for maintaining cell structure but also for recognizing various environmental signals, including hosts. Structurally, it comprises two layers: an inner layer containing a chitin/glucan matrix and an outer layer rich in glycoproteins [114]. Given that many glycosylated proteins are secreted or transported to the extracellular matrix, such as the membrane and cell wall, the study of cell wall glycoproteins is of particular interest in glycobiome research [23,24,26]. In our investigation of the *F. graminearum* cell wall, we observed that chitin was more abundant than glucose in both conidia and mycelia, although the glucose-to-glucosamine ratio differed. This finding aligns with the previous analysis of *F. graminearum* cell wall composition [115]. Furthermore, the previous research showing higher expression levels of chitin synthase in hyphae compared to germinating conidia supports our observation that glucosamine constitutes a larger proportion in mycelia than in conidia [116]. *O*-glycosylation plays a crucial role in maintaining fungal cell wall structure by regulating the abundance and distribution of key components such as *β*-1,3-glucans, chitin, and cell wall-associated glycoproteins [117]. In our phenotypic assays under cell wall stress conditions, several glycosyltransferase mutants exhibited altered sensitivity to agents such as Congo Red and SDS. These findings suggest that defects in glycosylation may influence or activate the cell wall integrity (CWI) signaling pathway, potentially through altered cell wall architecture or stress responses. Based on the analysis of cell wall components, we infer the presence of *N*- and *O*-glycan structures in cell wall glycoproteins. Given the conservation of the galactosyltransferase gene in *S. pombe* [118], *A. fumigatus* [119] and *T, reesei* [120,121], we investigated whether galactose exists as an *N*- or *O*- glycan in cell wall glycoproteins or simply as a component of the cell wall polysaccharides. Monosaccharide composition analysis of cell wall glycoproteins indicated that galactose is primarily present in cell wall polysaccharides rather than as carbohydrate moieties of glycoproteins. Galactose may be incorporated into various polysaccharide structures, such as *α*-1,4 poly-galacturonan, *β*-1,4 galactan, rhamno-galacturonan, arabinogalactan, galactoxylomannan, and galactomannan depending on the species [122–124]. In *Aspergillus* spp. [124], for example, galactomannan, composed of mannose and galactofuranose, is a cell wall component. Because UDP-galactopyranose mutase, an enzyme involved in galactofuranose biosynthesis, is conserved in *F. graminearum* [125]*,* and two distinct galactofuranose antigens (galactomannan and AB135–8 antigen) have been detected in *Fusarium* spp. [122], it is conceivable that galactose exists as galactomannan in *F. graminearum.*

Glycans play diverse biological functions, including protein folding, quality control, localization, and mediation of host-pathogen interactions. While all living organisms possess the protein glycosylation pathway, glycan processing varies significantly among species [126,127], leading to glycan diversity. Stage-specific glycan heterogeneity observed in various species underscores the biological significance of their structures, particularly in mammals [128–132]. However, the defined roles of glycan structures in glycoproteins, especially in processes like host-pathogen interactions, remain largely unexplored in plant pathogenic fungi. In this study, we provide the first comprehensive analysis of *N*- and *O*-glycan structures in phytopathogenic fungi *F. graminearum*. Intriguingly, we observed a predominance of hypermannosylated -type of *N*-glycans in mycelia compared to conidia while a more extended *O*-glycans in conidia than in mycelia, indicating developmental stage-dependent glycan generation. Therefore, our study on the structures and biosynthesis pathways of *N*- and *O*-glycans provides a foundational understanding of the critical roles that glycan structures play in the pathogenicity of plant pathogenic fungi, including *F. graminearum*. Furthermore, the future study on the delineation of novel or stage-specific glycan structures would highlight potential targets for future fungicide development.

*N*-glycosylation coordinates not only glycan biosynthesis but also protein stability through the ERQC system in various organisms [19,133,134]. Recent glyproteomics studies in plant pathogenic fungi, *M. oryzae* [24] and *G. graminicola* [26], showed that proteins involved in ERQC systems were highly *N*-glycosylated and influenced by *N*-glycosylation. Quantification of the *N*-glycoproteome in *F*. *graminearum* revealed significant changes in protein glycosylation levels during exposure to fungicide [23]. In *B. cinerea,* secreted glycoproteins were identified in protein *O*-mannosyltransferase mutants (*PMTs*) [21].

Glycoproteomic analyses focusing on the importance of initial core *N-*glycosylation have not yet been conducted in *F. graminearum.* In this study, our comparative glycoproteomic analysis between the wild type and glycosyltransferase deficient mutants revealed that truncated *N*-glycan structures, caused by defects in the early core *N*-glycosylation pathway, impact a wide range of global glycoproteins. Proteins with hydrolase or peptidase activities, predominantly found in the extracellular region, were highly affected in *alg3* (*fg26583*) and *alg12* (*fg03053*) deletion mutants. This disruption might affect protein functions, stability, and localization, ultimately influencing virulence.

Previous glycoproteomic studies have also demonstrated that secreted proteins, such as effectors, are affected by glycosylation. For example, *N*-glycosylation of Cnx1 was essential for effector secretion in *G graminicola* [26]. Additionally, proteins predicted to be highly *O*-glycosylated were more abundant in the secretomes of mutants with defects in *O*-glycosylation in *B. cinerea* [21]*.* This underscores the critical role of glycosylation in the biosynthetic-secretory pathway within the ER and Golgi apparatus. Western blotting analysis of three target glycoproteins previously implicated in virulence confirmed that they were under-glycosylated in *alg3* and *alg12* deletion mutants. Among these, Glx, identified as a highly conserved cellular surface antigen in *Fusarium* species, has been shown to play a dual role in regulating both virulence and mycotoxin biosynthesis by catalyzing the production of hydrogen peroxide from hosts [70]. Additionally, Cnb1 and Gic1, identified in previous *F. graminearum* phosphate study, exhibited reduced virulence upon deletion.

We also identified 34 essential or pathogenicity-associated glycoproteins that are impacted by aberrant glycans resulting from disruptions in initial glycan synthesis in *F. graminearum*. Among them, some glycoproteins were predicted to be localized in nucleus suggesting the possibility of *N*-glycosylation of nuclear proteins. Similarly, previous studies in mammal cells have reported the presence of *N*-glycosylated proteins in the nucleus, and these *N*-glycans play a critical role in regulating their activity [135–139]. Based on these observations, the transcription factors identified in this study are likely *N*-glycosylated in the nucleus or may be localized not only in the nucleus but also in the cytoplasm or other cellular regions in *F. graminearum*. In addition to transcription factors, we identified several virulence-related glycoproteins affected by aberrant glycosylation in *F. graminearum*, including phosphatases (Cna1, Pp2a, Gic1, Pho8, Psr2, and Fg07304), kinases (Ste7 and Cmk1), a peroxidase (Fca7), 70-kDa heat shock proteins (Kar, Ssc, and Sse), a lipase (Tag), chitin synthases (Chs5 and Chs7), autophagy-related proteins (Rab11, Vps74, and Atg27), a G protein (Gpb1), an E3 ubiquitin ligase (Skp1), and an adenylate cyclase-associated protein (Cap1), which is required for the activation of adenylate cyclase and cAMP signaling. Additional affected proteins include cytidylyltransferase (Ptc1), which plays a role in phospholipid biosynthesis; aldehyde dehydrogenase (Aldh); an ATP-binding cassette (ABC) transporter (Arb1); a subtilisin-like protease (Prb1); and acetohydroxyacid synthases (Ilv6), which are involved in branched-chain amino acid biosynthesis. Other glycoproteins affected include Cfemn1, Ade5, Erg9, Syn2, Glx, and Tub2.

Among these, only the peroxidase KatG2 (also known as Fca7) has been previously reported to be *N*-glycosylated at asparagine residues at positions 238 and 391, both of which are essential for its accumulation and cell wall localization in planta in *F. graminearum* [94]. More detailed studies in other fungal pathogens support the critical role of proper glycosylation in regulating protein stability and pathogenicity. For example, in *G. graminicola*, the *N*-glycosylated effector protein Nis1 was shown to degrade significantly faster in the Δ*alg3* mutant than in the wild-type strain indicating that disruption of the *N*-glycosylation pathway compromises protein stability and processing [26]. Similarly, in *C. neoformans*, proper extension of *O*-mannosylation in the Golgi apparatus is essential for host-pathogen interactions throughout disease progression—from adherence to lung epithelial cells and survival within macrophages to transmigration across the blood-brain barrier [16]. Furthermore, in *M. oryzae*, *N*-glycosylation is required not only for maintaining the ER quality control system during development and pathogenesis, but also for ensuring the stability and chitin-binding activity of the effector protein Slp1 [20,24]. Collectively, these findings suggest that proper glycosylation of virulence-associated proteins is crucial for their stability, trafficking, and function. Therefore, further investigation is needed to elucidate how glycan truncation disrupts the biological activity, localization, or secretion of these glycoproteins and thereby contributes to reduced virulence in *F. graminearum*. A more detailed understanding of these mechanisms may reveal potential targets for antifungal strategies aimed at disrupting glycosylation-dependent pathogenicity.

In summary, we propose a working model of how *N*-glycosylation modulates the structure of *N-*glycans attached to global glycoproteins and affects fungal virulence (Fig 8). These findings indicate that aberrant glycan structures of cell wall glycoproteins, critical to *F. graminearum* virulence, could generate dramactically decreased virulence. Further investigations are required to elucidate the underlying mechanisms how the truncated glycan structures affect virulence by investigating the altered function, stability, and localization of virulence-associated glycoproteins [38]. Additionally, studies focusing on upstream regulators that coordinate the expression of glycosylation-related genes are essential for a comprehensive understanding of the protein glycosylation process.

In conclusion, this systematic functional profiling of protein glycosylation enhances our understanding of the complex roles of glycosylation, highlighting the connections among genes involved in the protein glycosylation pathway, glycans, and glycoproteins in regulating the general biology and pathogenicity of *F. graminearum*.

## Supporting information

S1 Text Fig A in S1 Text: Confirmation of gene involved in protein glycosylation deletion mutants by Southern blot analysis. **Fig B in S1 Text:** Phenotypic traits of genes involved in protein *N*, *O*-glycosylation at the ER (endoplasmic reticulum) and Golgi (golgi apparatus). **Fig C in S1 Text:** Spearman’s rank correlation among multiple phenotypes was calculated for each mutant phenotype. **Fig D in S1 Text:** Transcript levels of genes involved in protein glycosylation across these stages. **Fig E in S1 Text:** Phenotype of deletion mutants under various stress conditions. **Fig F in S1 Text:** Spearman’s rank correlation among multiple phenotypes under various stress conditions was calculated for each mutant phenotype. **Fig G in S1 Text:** Sample preparation for glycoproteomic analysis. **Fig H in S1 Text:** PCA analysis for glycoproteomic samples. **Fig I in S1 Text:** Prediction of subcellular localization and functional classification of identified glycoproteins. **Fig J in S1 Text:** Pearson correlation analysis between whole-cell extracts and glycoprotein samples in the wild type, *fg03053*, and *fg26583* deletion mutants.
(DOCX)

S1 Table Primers used in this study. (XLSX)

S2 Table Comparative analysis of protein glycosylation-related genes among fungal species. (XLSX)

S3 Table Collection of phenotypic analyses for 65 deletion mutants involved in protein glycosylation in *F. graminearum.* (XLSX)

S4 Table Transcript levels of genes involved in protein glycosylation across these stages in *F. graminearum.* (XLSX)

S5 Table In vitro phenotypic traits under 20 different growth conditions. (XLSX)

S6 Table Monosaccharide components analysis in cell wall and glycoprotein in *F. graminearum.* (XLSX)

S7 Table Identified glycoproteins in *F. graminearum.* (XLSX)
