# Supplementary material for: Integrative glycomic analysis reveals the crucial role of protein glycosylation in fungal pathogenesis
Source: PLoS Pathog. 2025 Jul 7;21(7):e1013325. doi: 10.1371/journal.ppat.1013325 (PMC12244721; doi:10.1371/journal.ppat.1013325)
Supplement: S1 Text — Fig B in S1 Text: Phenotypic traits of genes involved in protein N, O-glycosylation at the ER (endoplasmic reticulum) and Golgi (golgi apparatus). Fig C in S1 Text: Spearman’s rank correlation among multiple phenotypes was calculated for each mutant phenotype. Fig D in S1 Text: Transcript levels of genes involved in protein glycosylation across these stages. Fig E in S1 Text: Phenotype of deletion mutants under various stress conditions. Fig F in S1 Text: Spearman’s rank correlation among multiple phenotypes under various stress conditions was calculated for each mutant phenotype. Fig G in S1 Text: Sample preparation for glycoproteomic analysis. Fig H in S1 Text: PCA analysis for glycoproteomic samples. Fig I in S1 Text: Prediction of subcellular localization and functional classification of identified glycoproteins. Fig J in S1 Text: Pearson correlation analysis between whole-cell extracts and glycoprotein samples in the wild type, fg03053, and fg26583 deletion mutants. (DOCX) [file ppat.1013325.s001.docx]

**Supplementary Information**

**Integrative glycomic analysis reveals the crucial role of protein glycosylation in fungal pathogenesis**

Heeji Moon^1†^, Eun Jung Thak^2,3†^, Yejin Choi^1^, Sieun Kim^4^, Jiyeun Park^5^, Nahyun Lee^1^, Soobin Shin^1^, Hosung Jeon^1^, Jessica Winarto^6^, Soyoung Choi^1^, Ji Young Shin^7^, Jung-Eun Kim^8^, Dae-Geun Song^6,9^, Hun Kim^10^, Gyung Ja Choi^10^, Hyun Ah Kang^2*^, and Hokyoung Son^1,11,12,13*^

*^1^Department of Agricultural Biotechnology, Seoul National University, Seoul, 08826, Republic of Korea*

*^2^Department of Life Science, Chung-Ang University, Seoul, 06974, Republic of Korea*

*^3^College of Pharmacy, Dongduk Women's University, Seoul, South Korea*

*^4^Horticultural and Herbal Crop Environment Division, National Institute of Horticultural and Herbal Science, Wanju, South Korea.*

*^5^Institute for Plant Sciences, University of Cologne, Cologne, Germany.*

*^6^Center for Natural Product Systems Biology, Korea Institute of Science and Technology (KIST) Gangneung Institute of Natural Products, Gangneung, 25451, Republic of Korea*

*^7^Division of Bioresources Bank, Honam National Institute of Biological Resources, Mokpo, 58762, Republic of Korea*

*^8^Research Institute of Climate Change and Agriculture, National Institute of Horticultural and Herbal Science, Rural Development Administration, Jeju, 63240, Republic of Korea*

*^9^Natural Product Applied Science, KIST School, University of Science and Technology, Gangneung, 25451, Republic of Korea*

*^10^Therapeutic & Biotechnology Division, Center for Eco-friendly New Materials, Korea Research Institute of Chemical Technology, Daejeon, 34114, Republic of Korea.*

*^11^Research Institute of Agriculture and Life Sciences, Seoul National University, Seoul, 08826, Republic of Korea*

*^12^Plant Genomics and Breeding Institute, Seoul National University, Seoul, 08826, Republic of Korea*

*^13^Plant Health Center, Seoul National University, Seoul 08826, Republic of Korea*

^†^These authors contributed equally to this work and share first authorship.

^*^Corresponding authors:

Hyun Ah Kang, hyunkang@cau.ac.kr

Hokyoung Son, hogongi7@snu.ac.kr


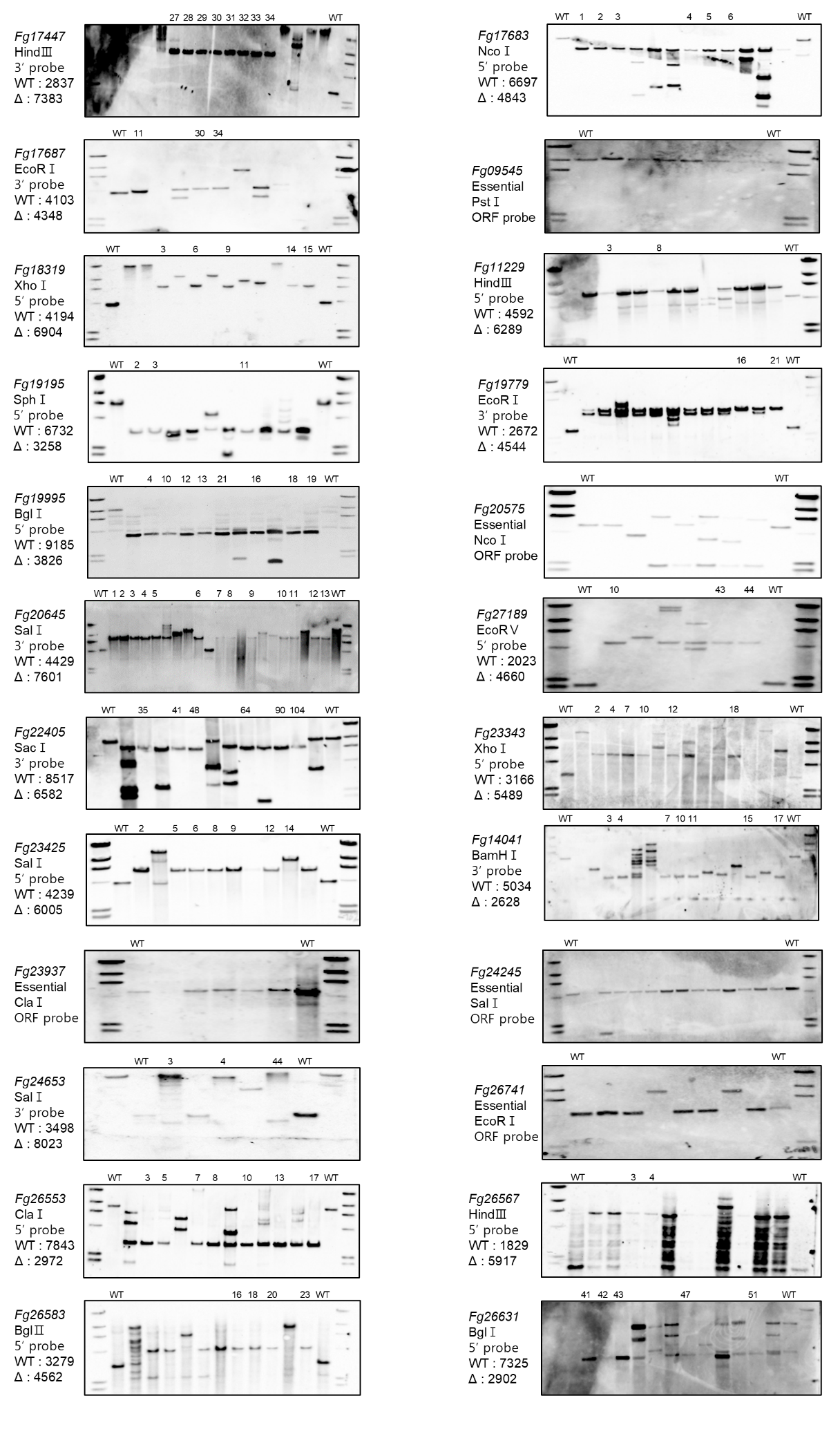

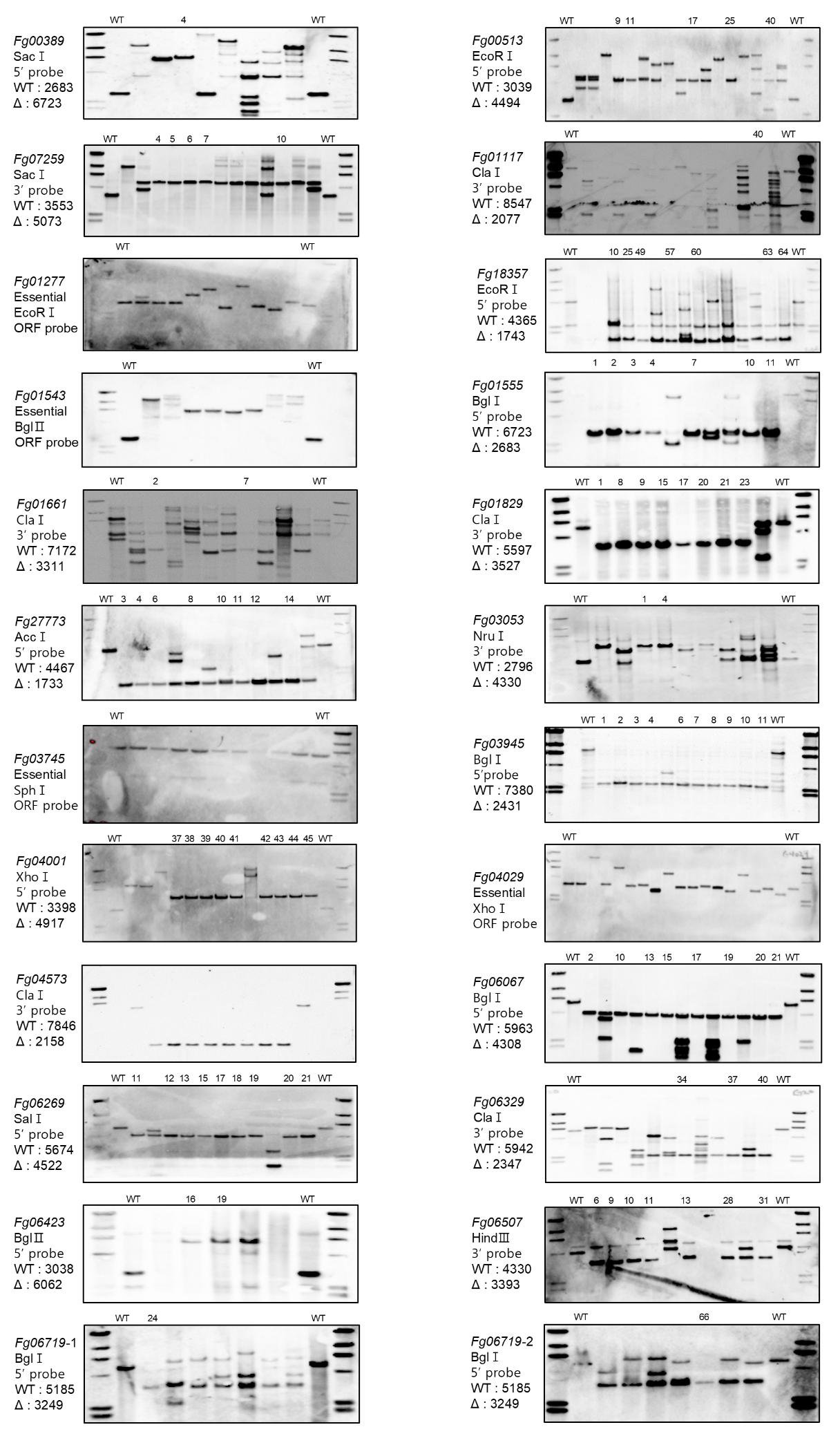

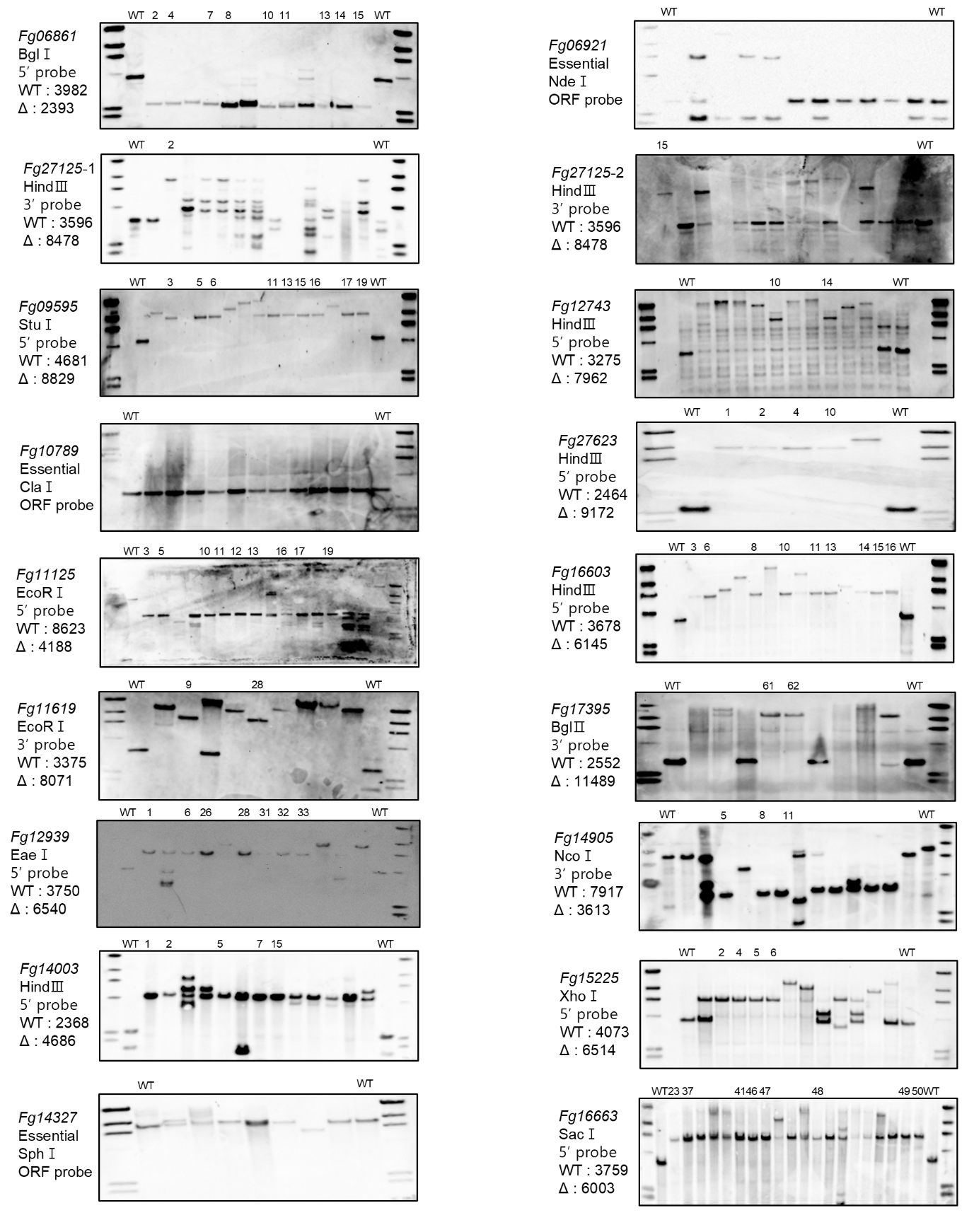


**Figure A. Confirmation of gene involved in protein glycosylation deletion mutants by Southern blot analysis.** The strain name, restriction enzymes used for each blot, probe and the size of the DNA standards (kb) are indicated on the left of each blot. WT, *F. graminearum* wild-type strain Z-3639.


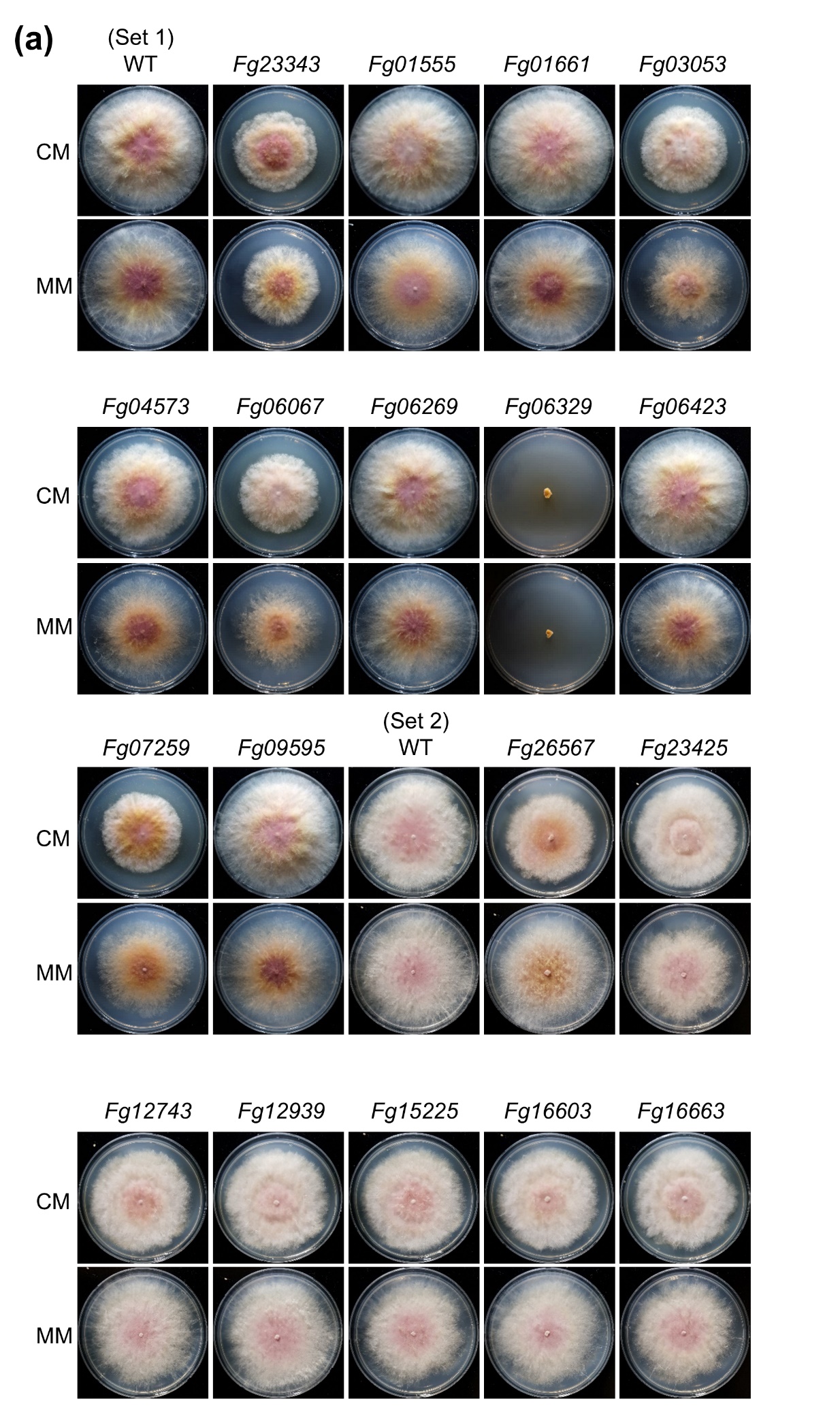


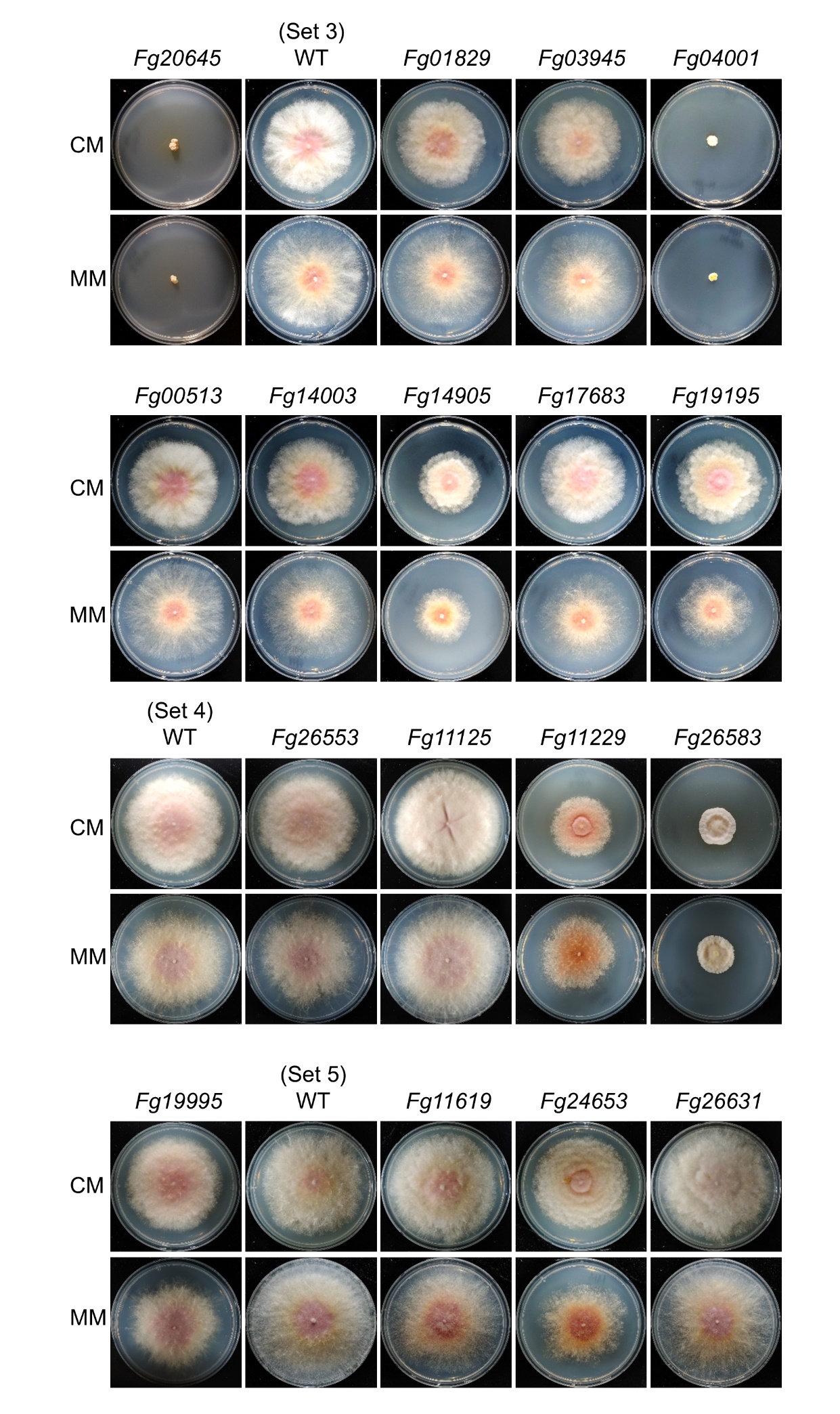

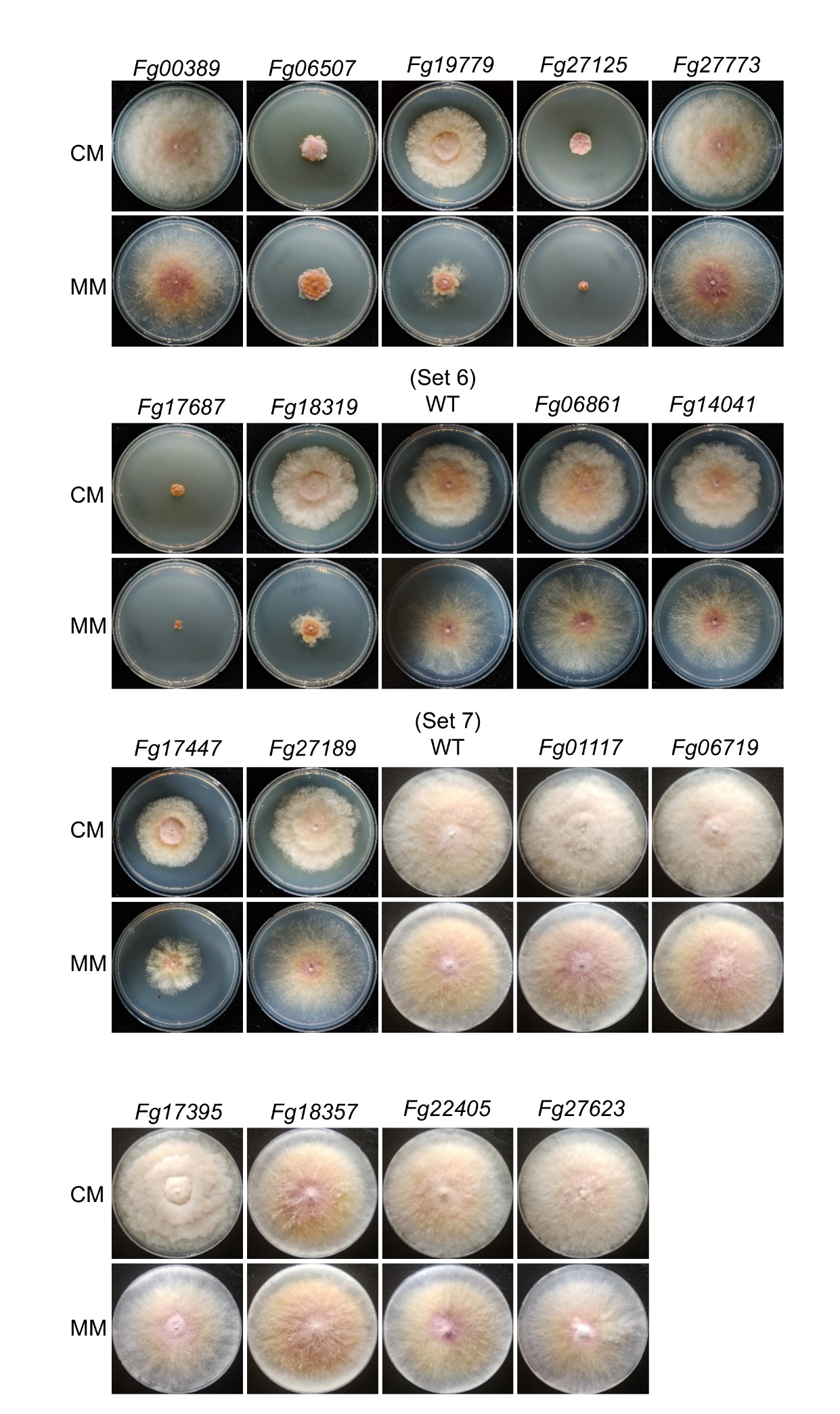


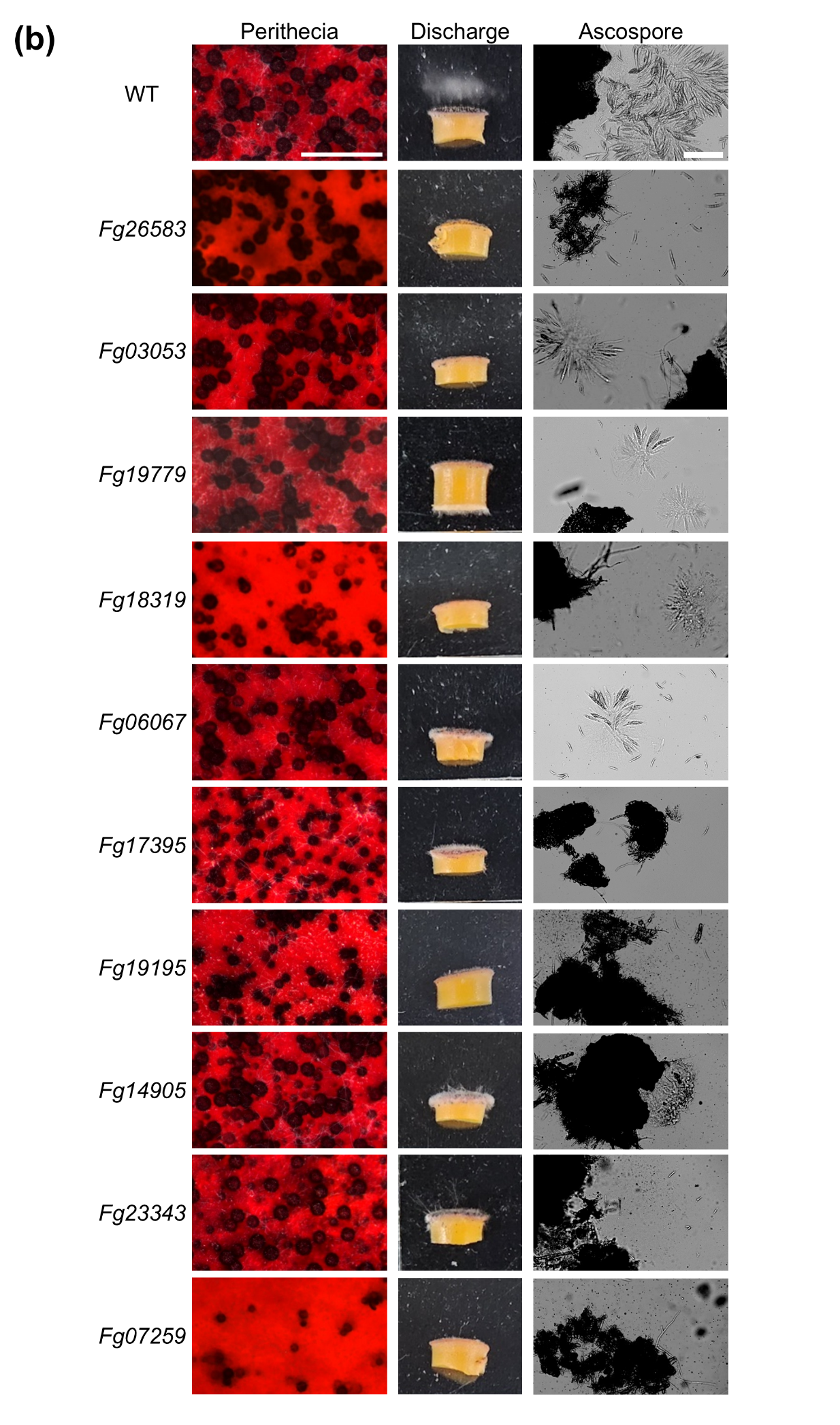


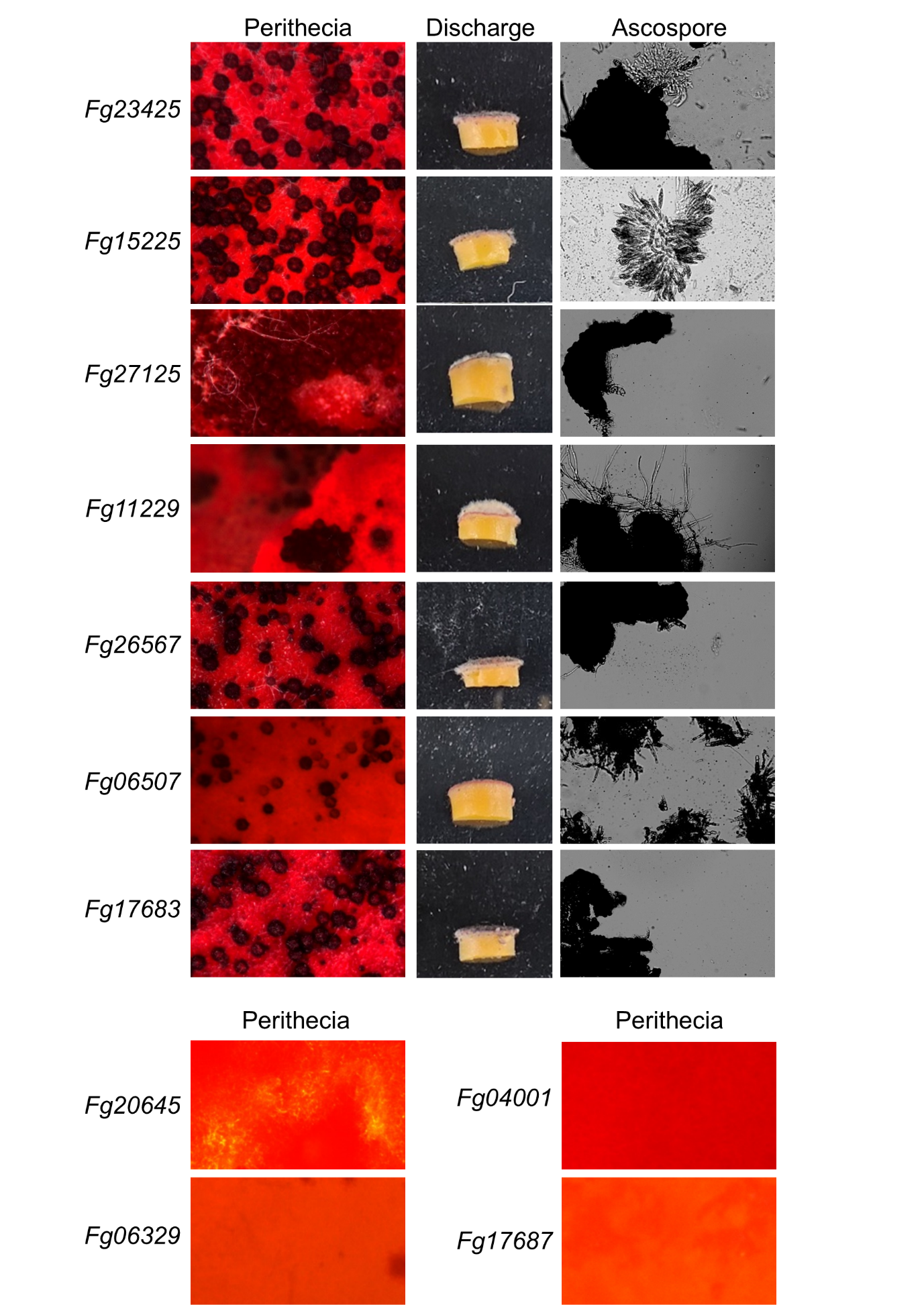


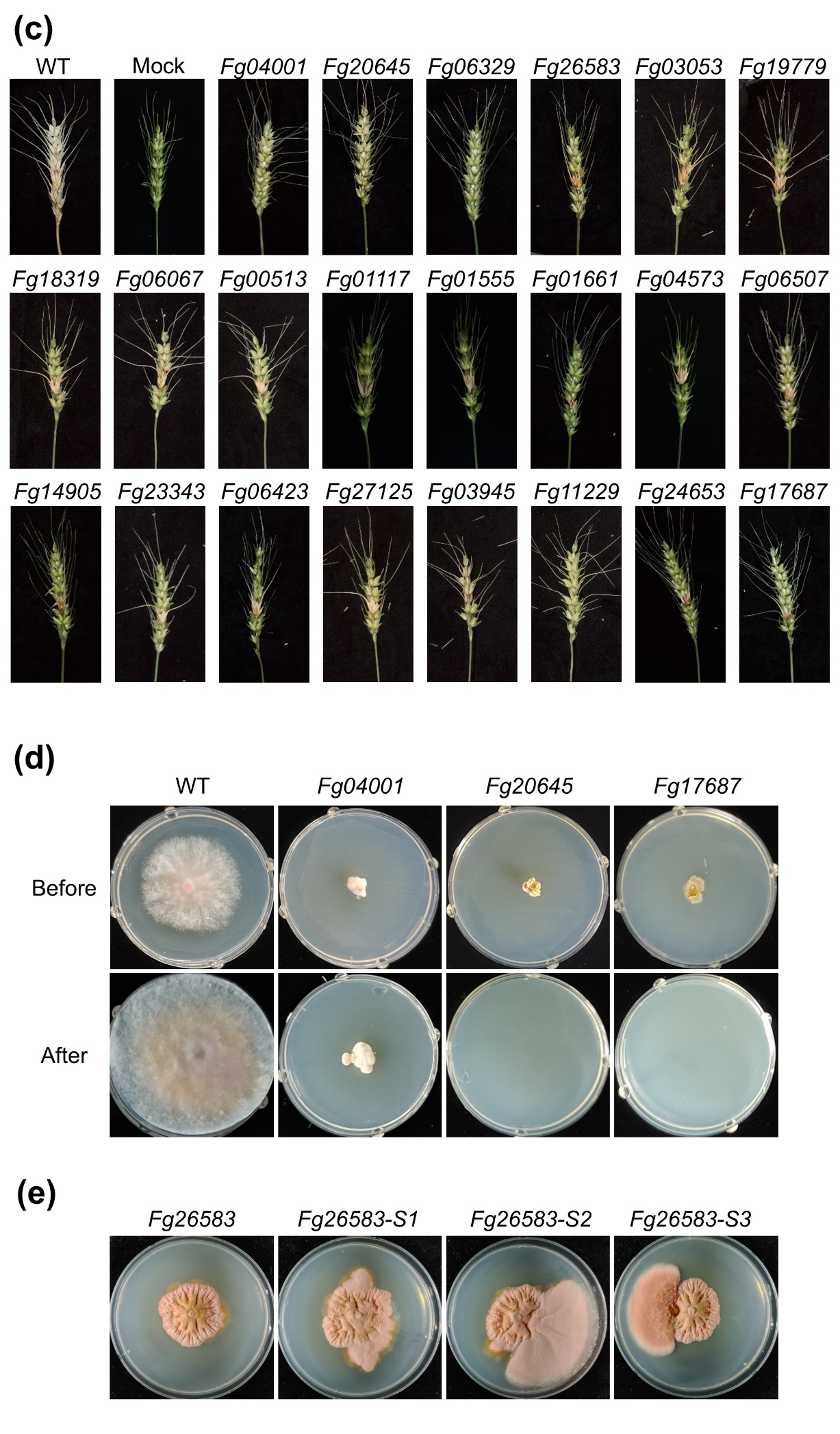


**Figure B. Phenotypic traits of genes involved in protein *N*, *O*-glycosylation at the ER (endoplasmic reticulum) and Golgi (golgi apparatus)** (a) Vegetative growth at minimal media (MM) and complete media (CM), Fungal strains were grown on MM for 5~6 days. WT, *F. gramieanrum* wild-type strain *Z3639*; (b) Sexual development, each strain was inoculated on carrot agar. The photographs were taken 10 days after sexual induction. WT, *F. gramieanrum* wild-type strain *Z3639,* Scale bar = 1 mm (dissecting microscope images) and 100 µm (differential interference contrast images); (c) Virulence of mutants on wheat heads, A center spikelet of each wheat head was injected with 10 µl of conidia suspension. The photographs were taken 14 days after inoculation. WT, *F. gramieanrum* wild-type strain *Z3639*.; (d) Cellophane penetration assays of deletion mutants. Fungal colonies were grown for 2 days at 25 °C on top of cellophane membranes placed on CM plates, then removed cellophane membranes with the fungal colonies, and the plates were incubated for an additional day to examine the presence of mycelial growth on the plate, indicating penetration of the cellophane. (e) Spontaneous suppressors of the *Fg26583* mutant. Sectors with faster growth rate were observed in CM media of the *Fg26583* mutant grown on 5~6 days.


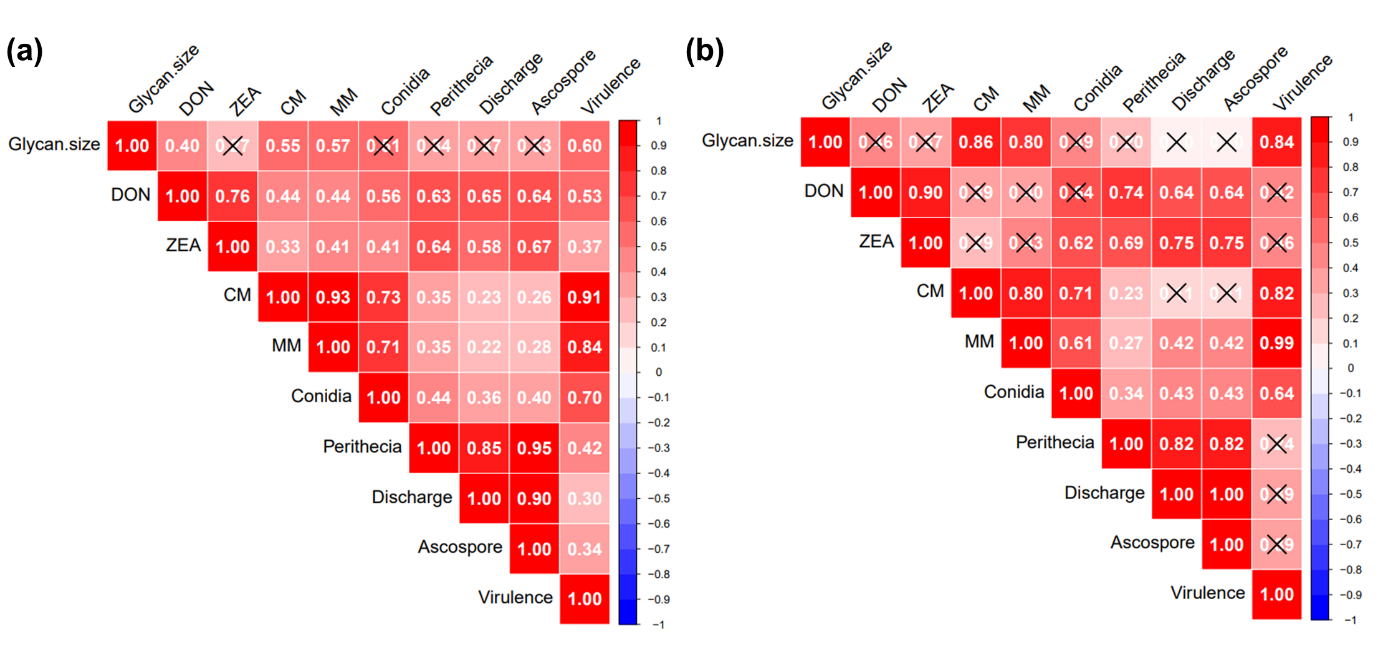


**Figure C. Spearman's rank correlation among multiple phenotypes was calculated for each mutant phenotype.** ‘X’ indicates that there is no significant correlation between the two phenotypes (*P* < 0.05; two-tailed *t*-test). The size of each glycan was manually determined based on the number of mannose and glucose residues, using the diagrams of the glycosylation pathway in *Saccharomyces cerevisiae* shown in Fig. 2a and 3a. (a) Genes involved in *N-*glycosylation, (b) Genes involved in *O*-glycosylation.

**
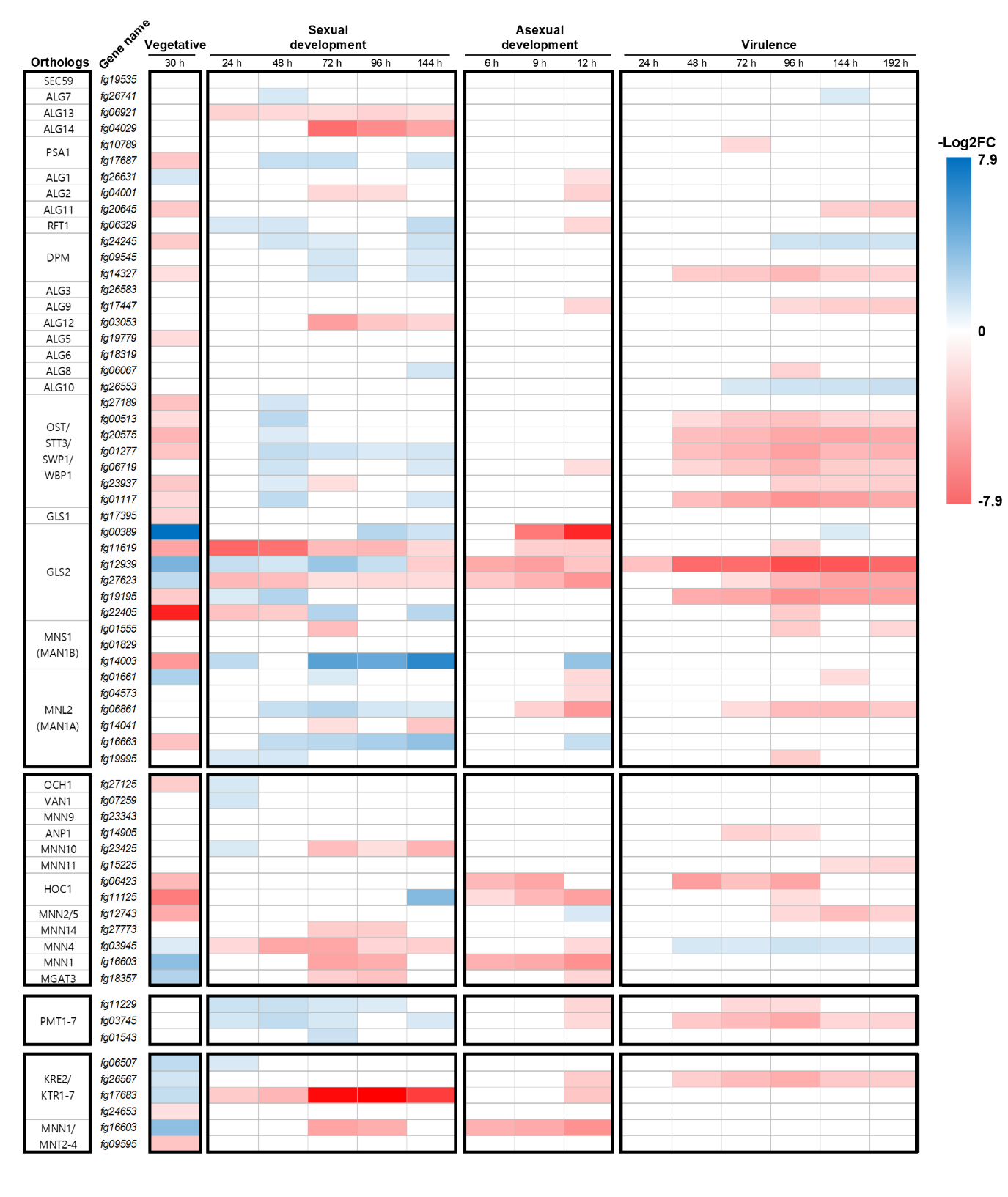
**

**Figure D. Transcript levels of genes involved in protein glycosylation across these stages.** Heatmap visualization of glycosylation involved genes transcriptional profiles during vegetative growth, virulence, asexual, and sexual development. The heatmap depicts gene transcript levels during vegetative growth, virulence, asexual, and sexual developmental time points based on Log2-based relative transcript abundances compared with 0 h conidia (vegetative growth and virulence), 3 h (asexual development) and 2 h (sexual development). Red represents higher expression; blue represents lower expression; and the rows represent transcriptional units. RNA-seq results were obtained from previous studies[1-4].

**
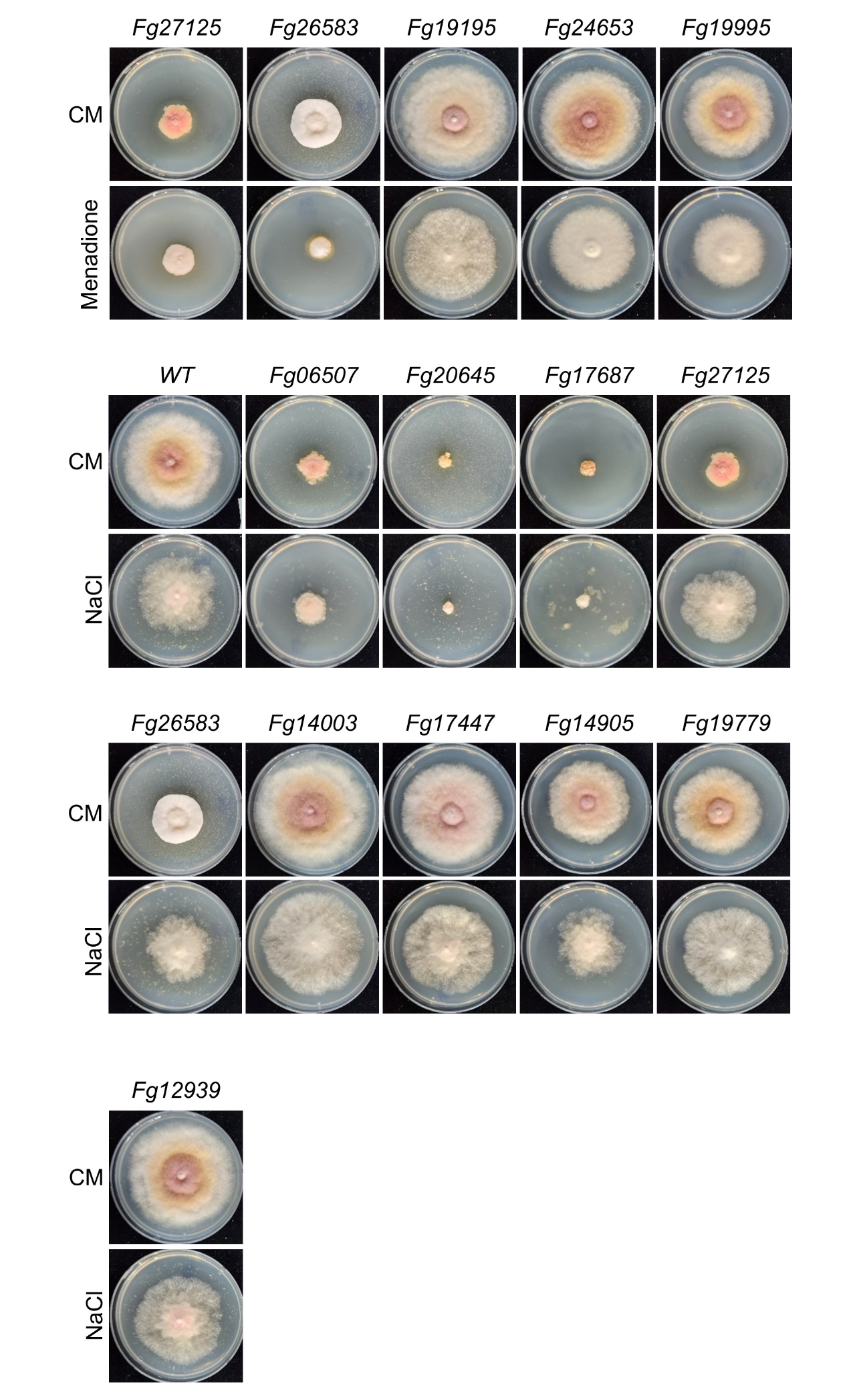

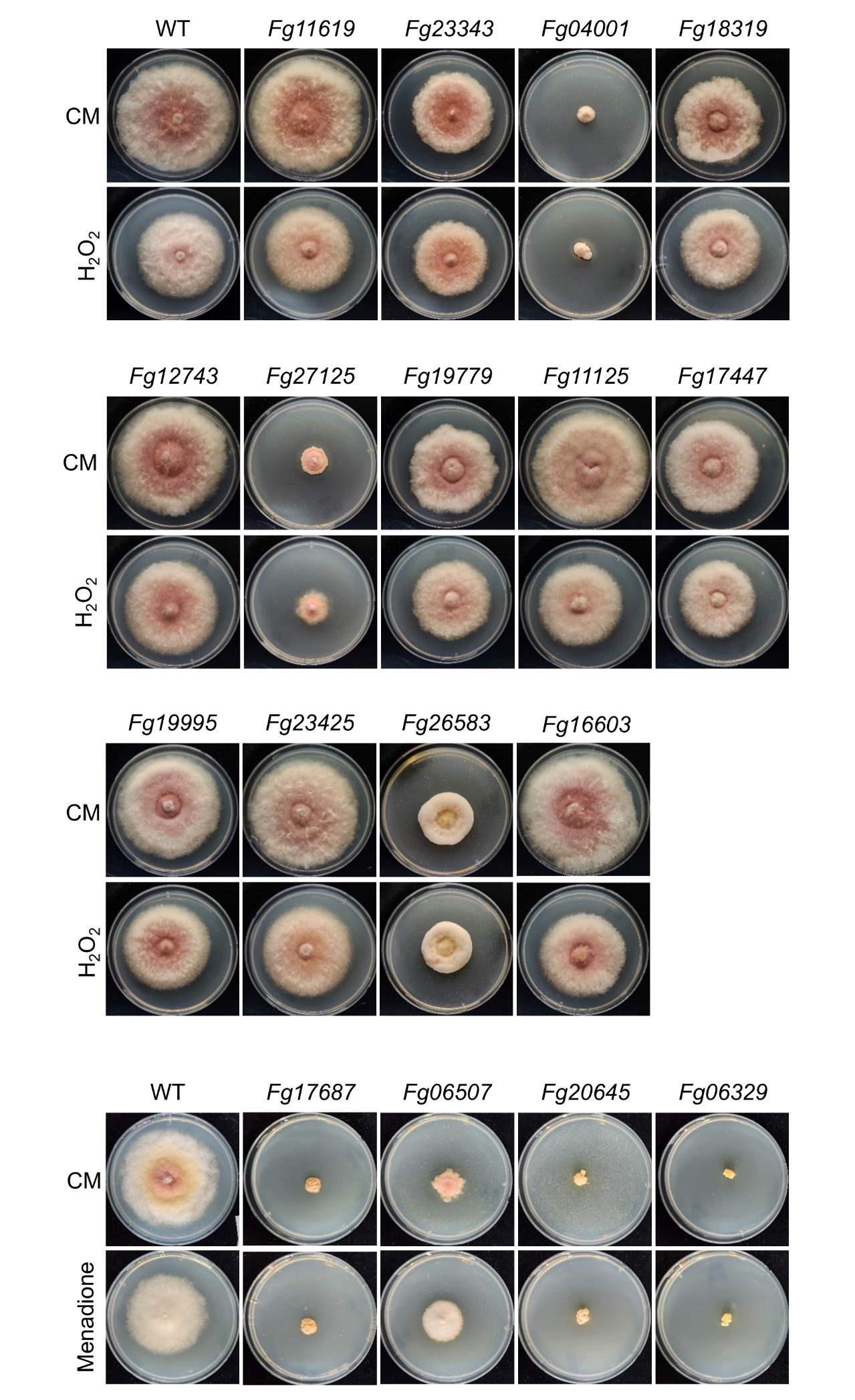

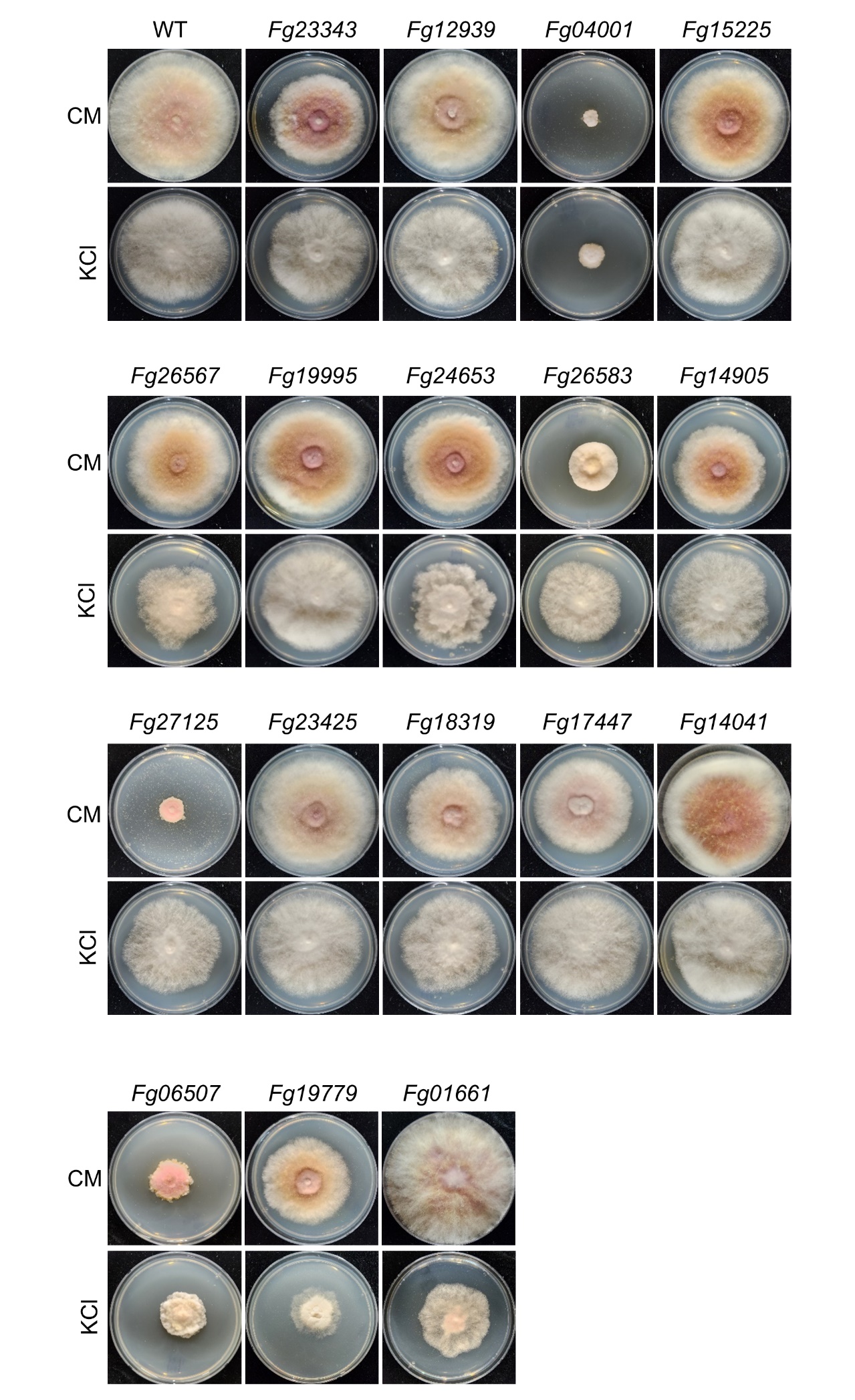

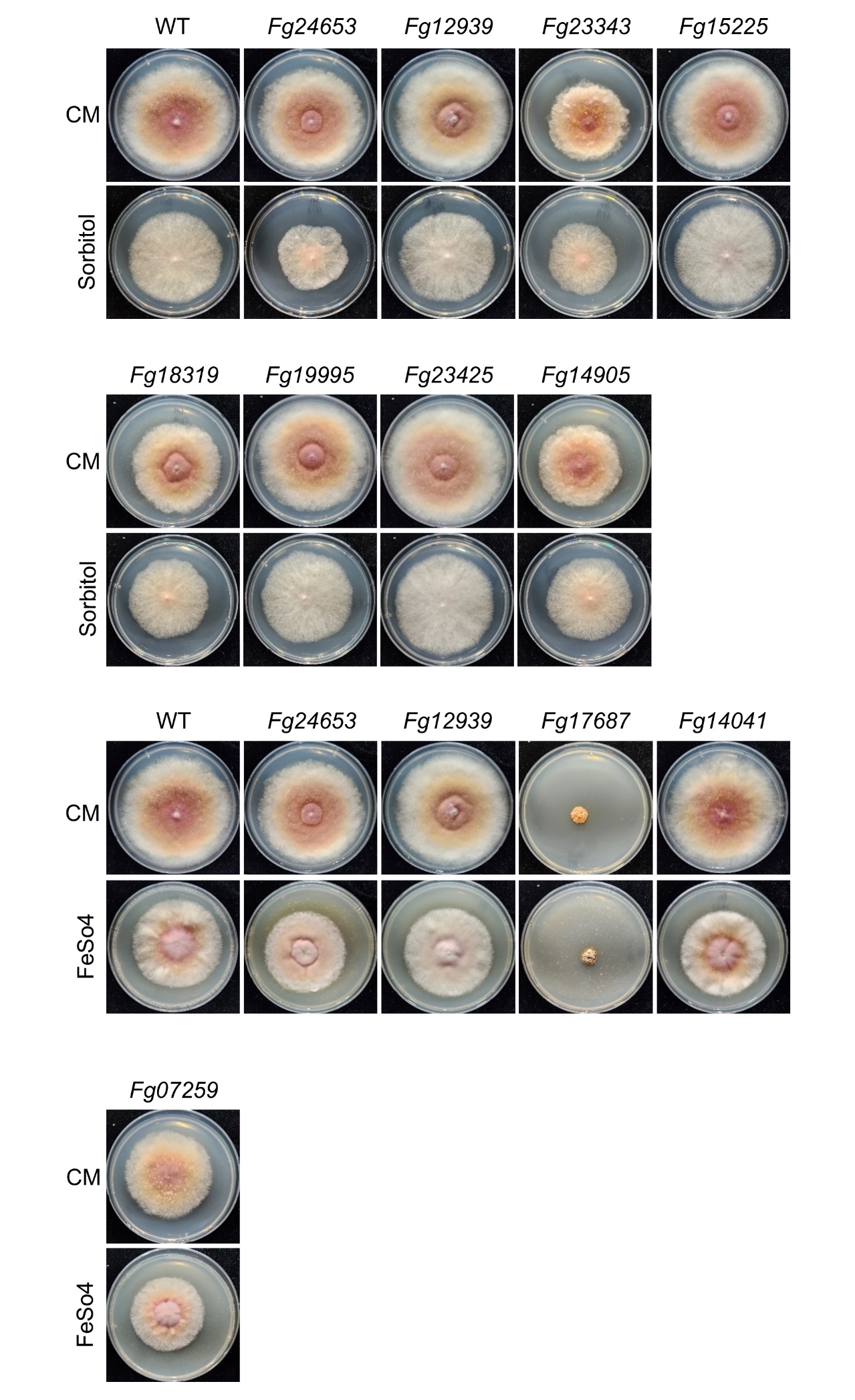

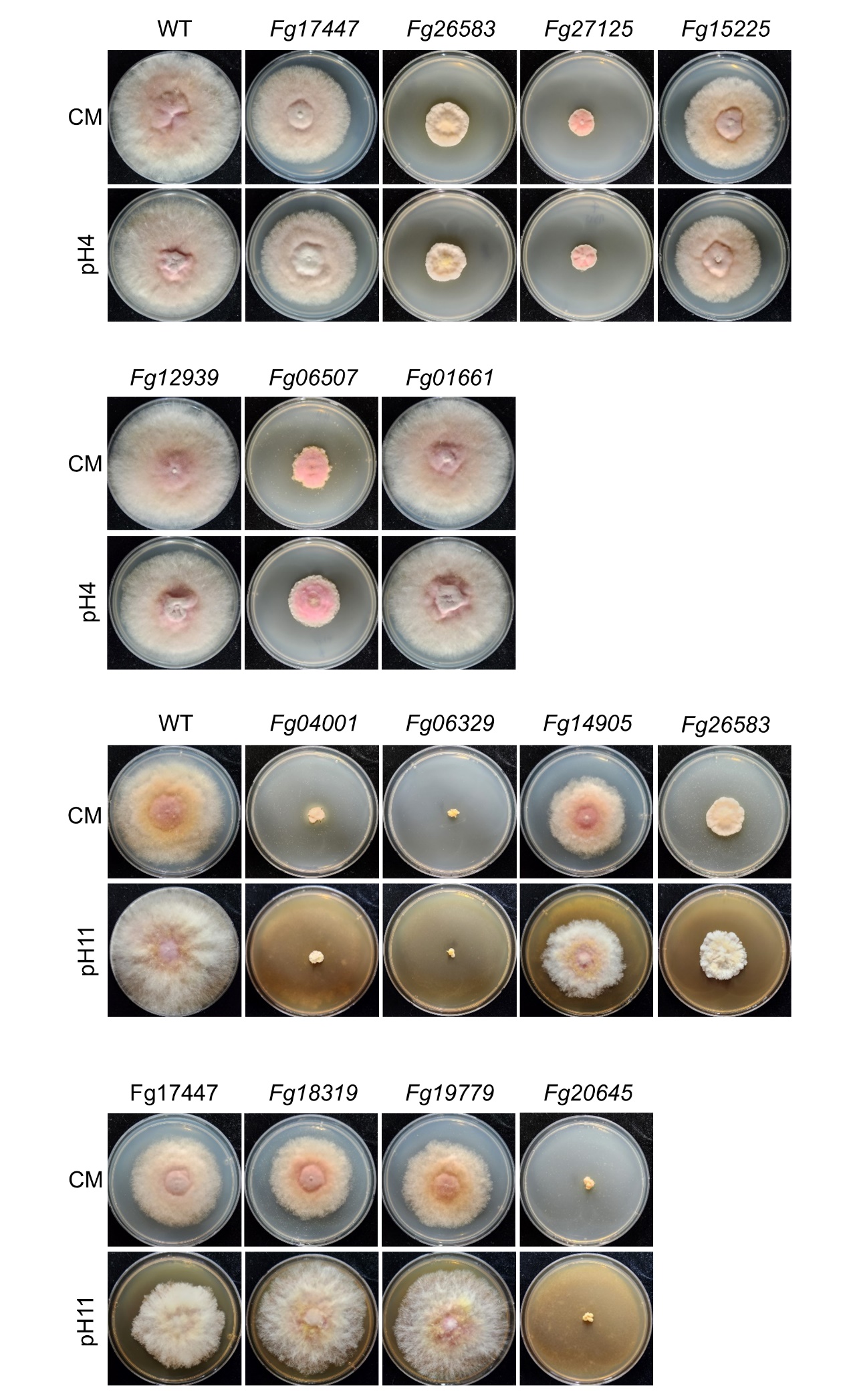

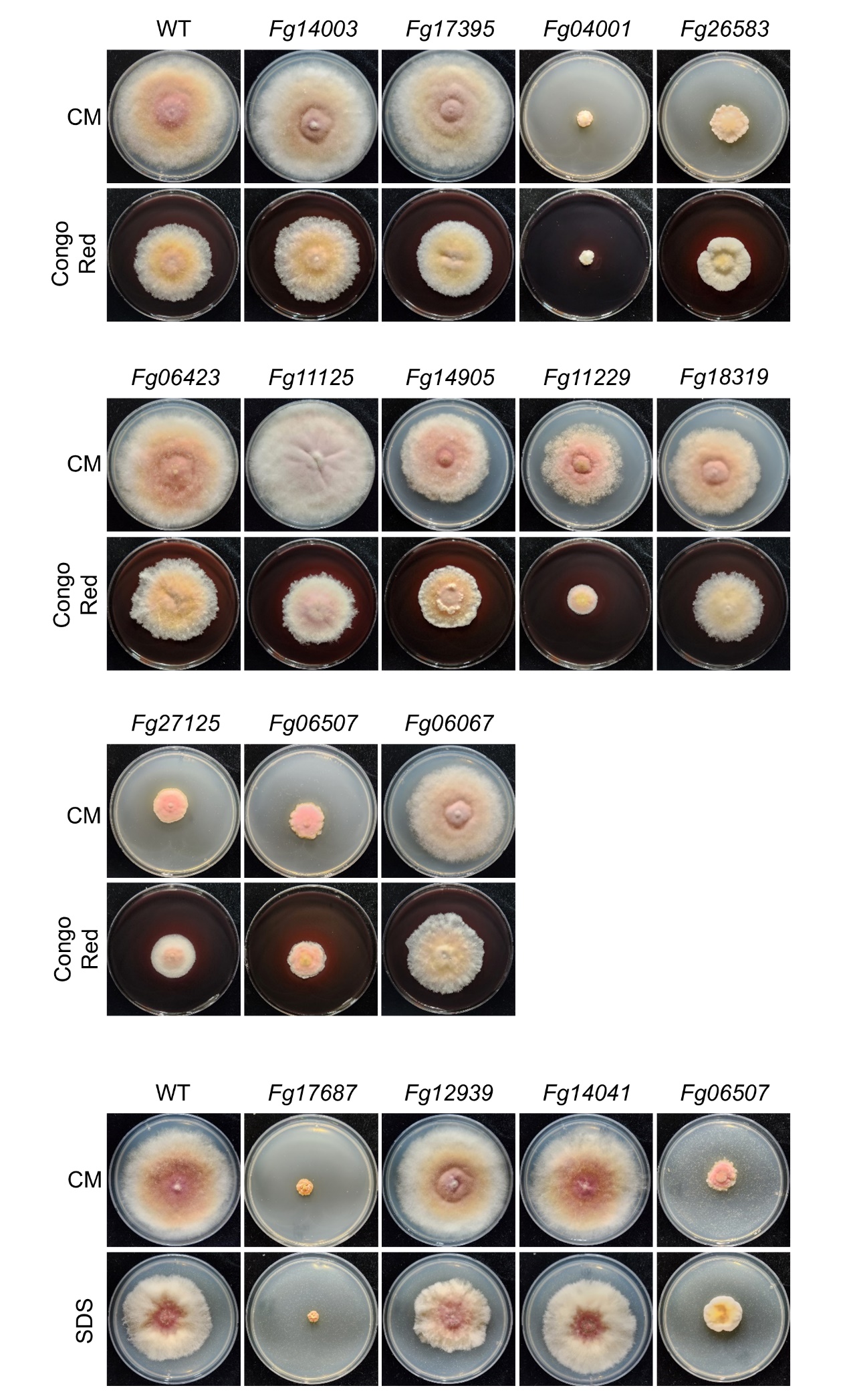

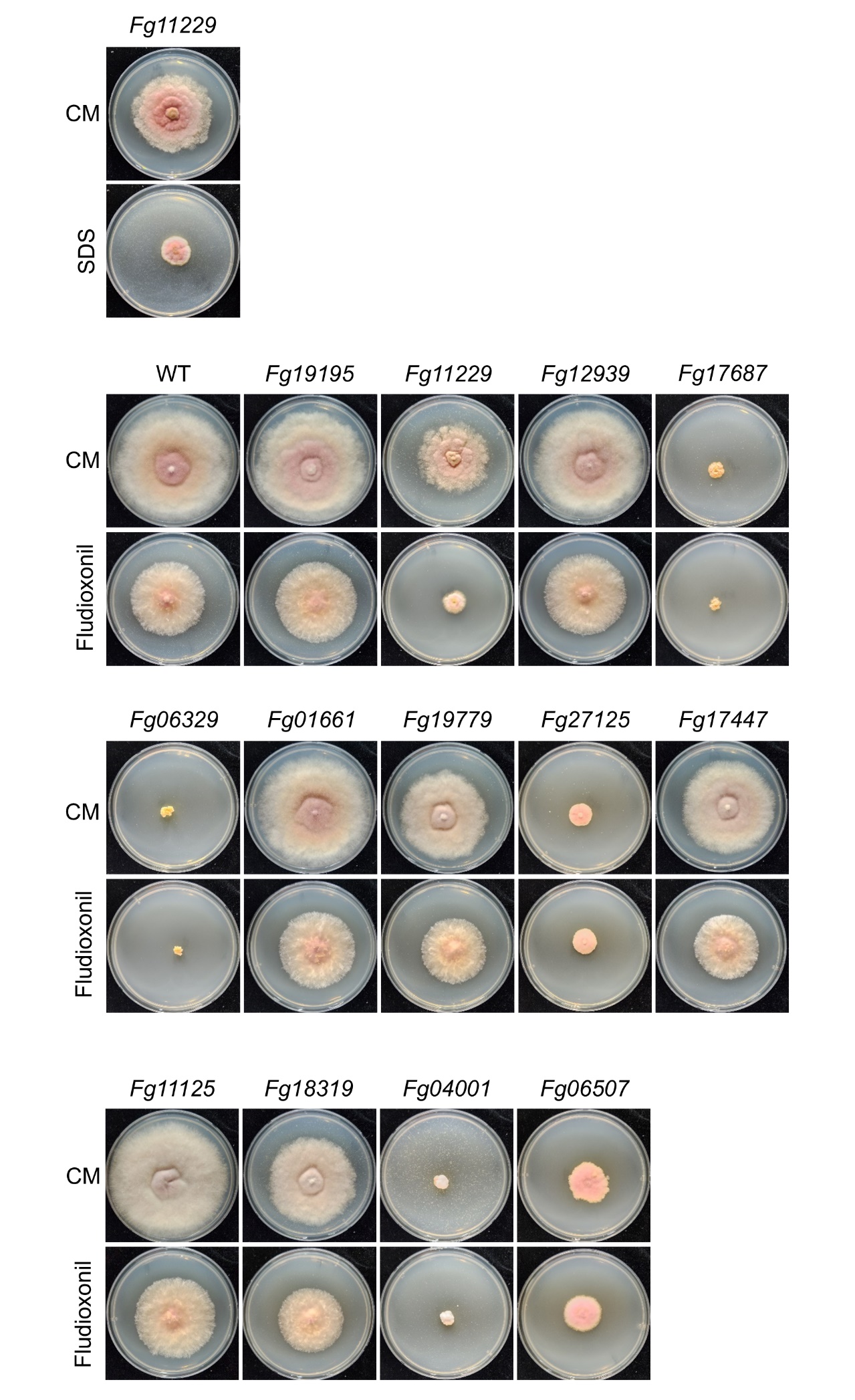

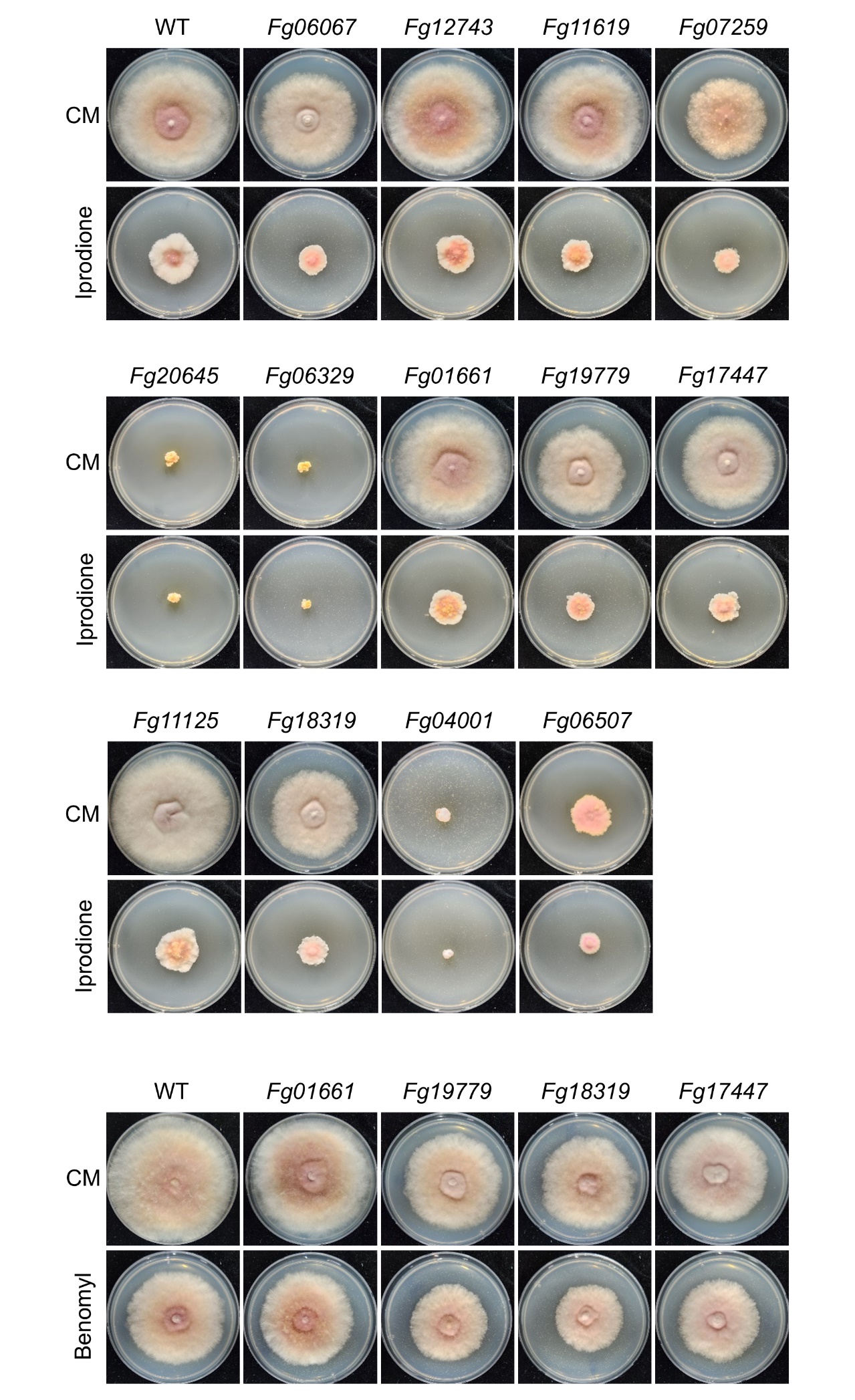

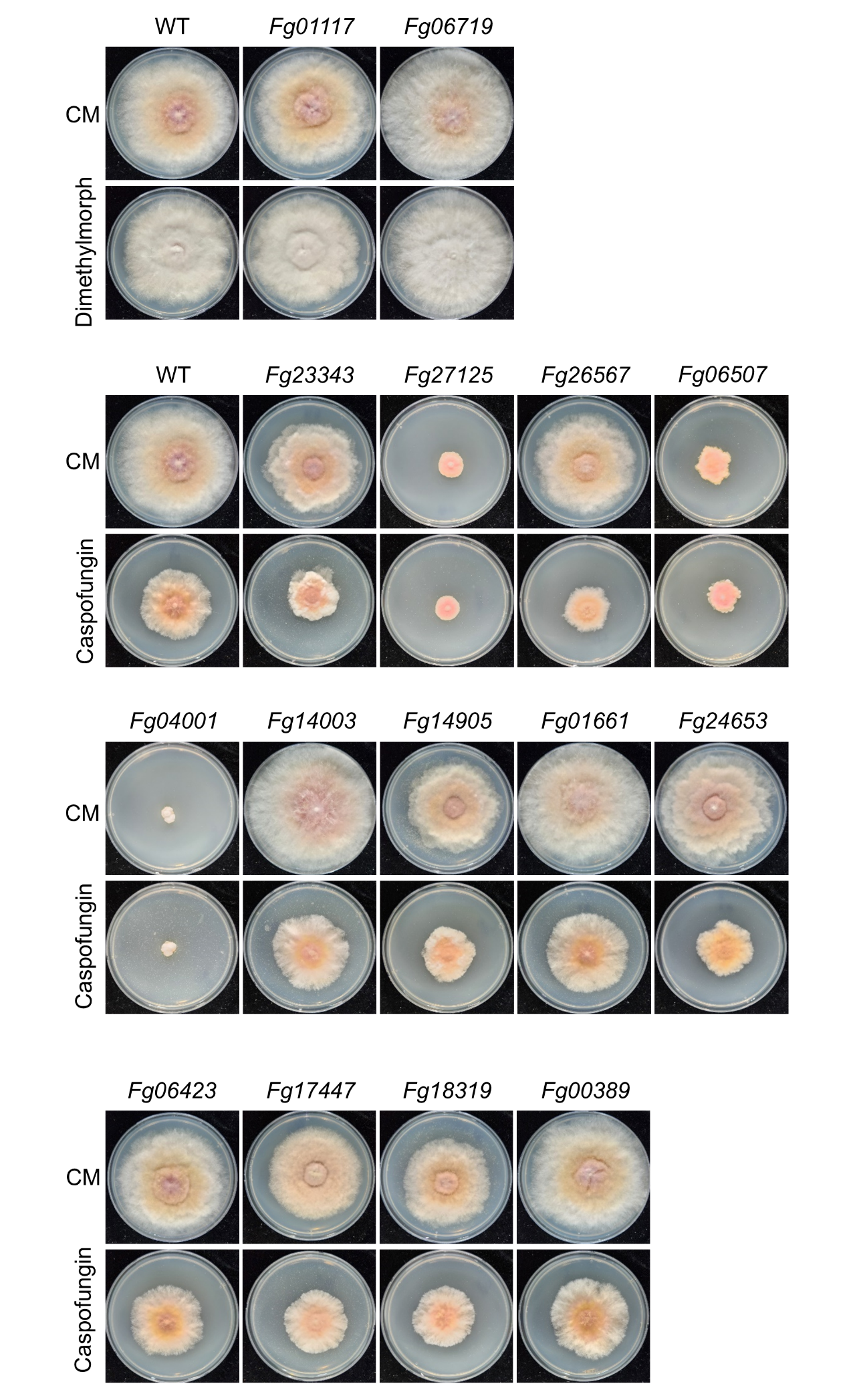

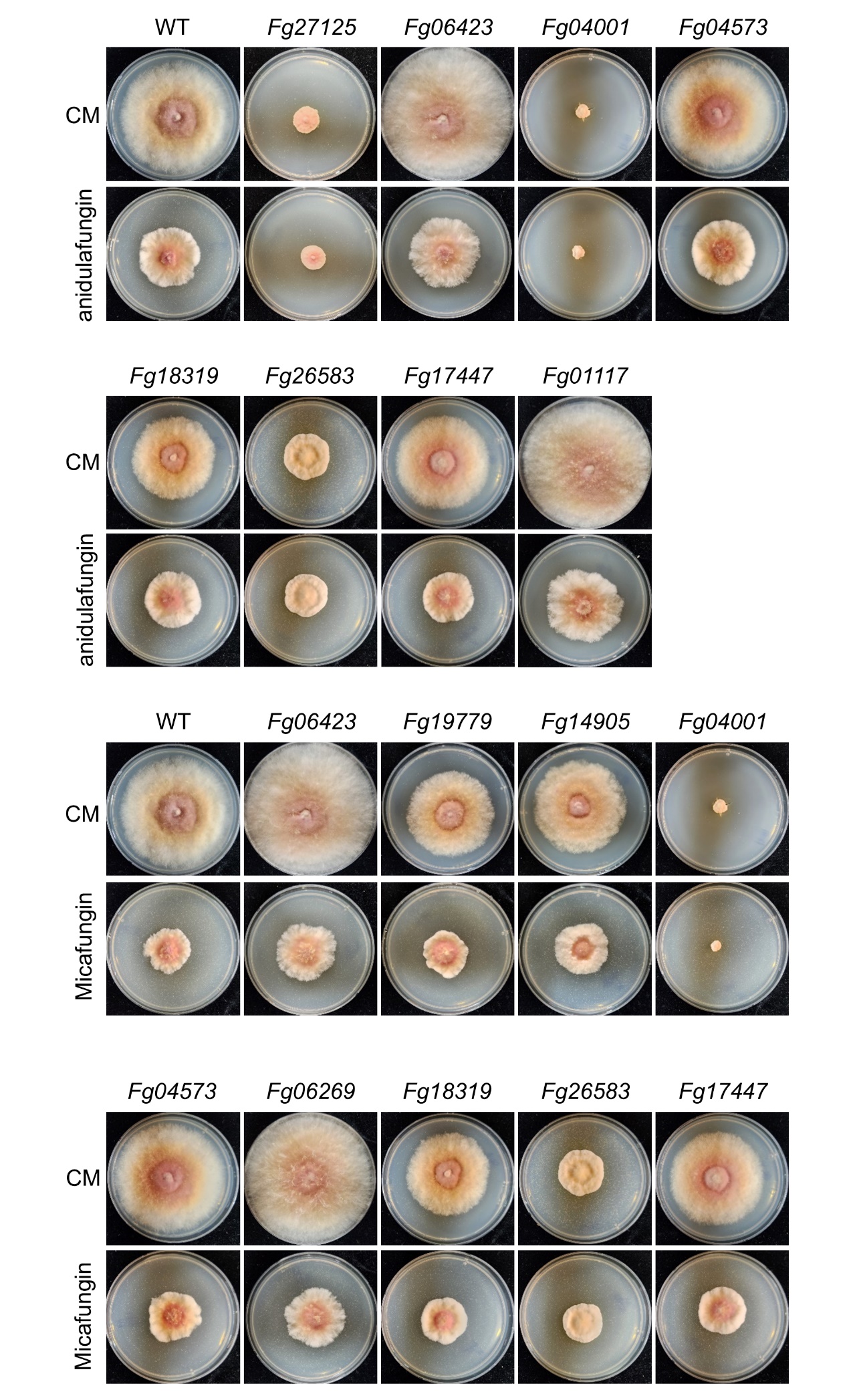

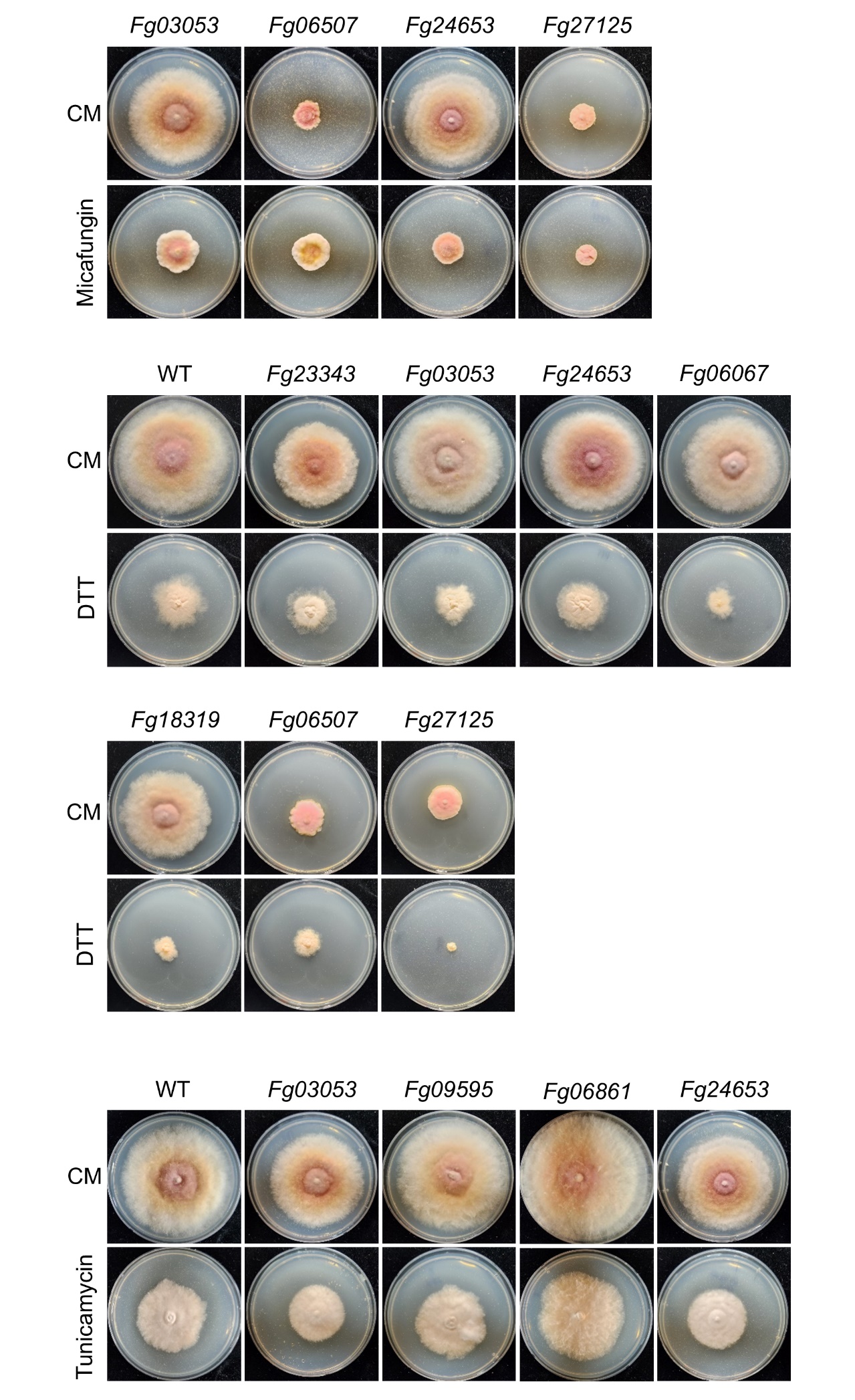

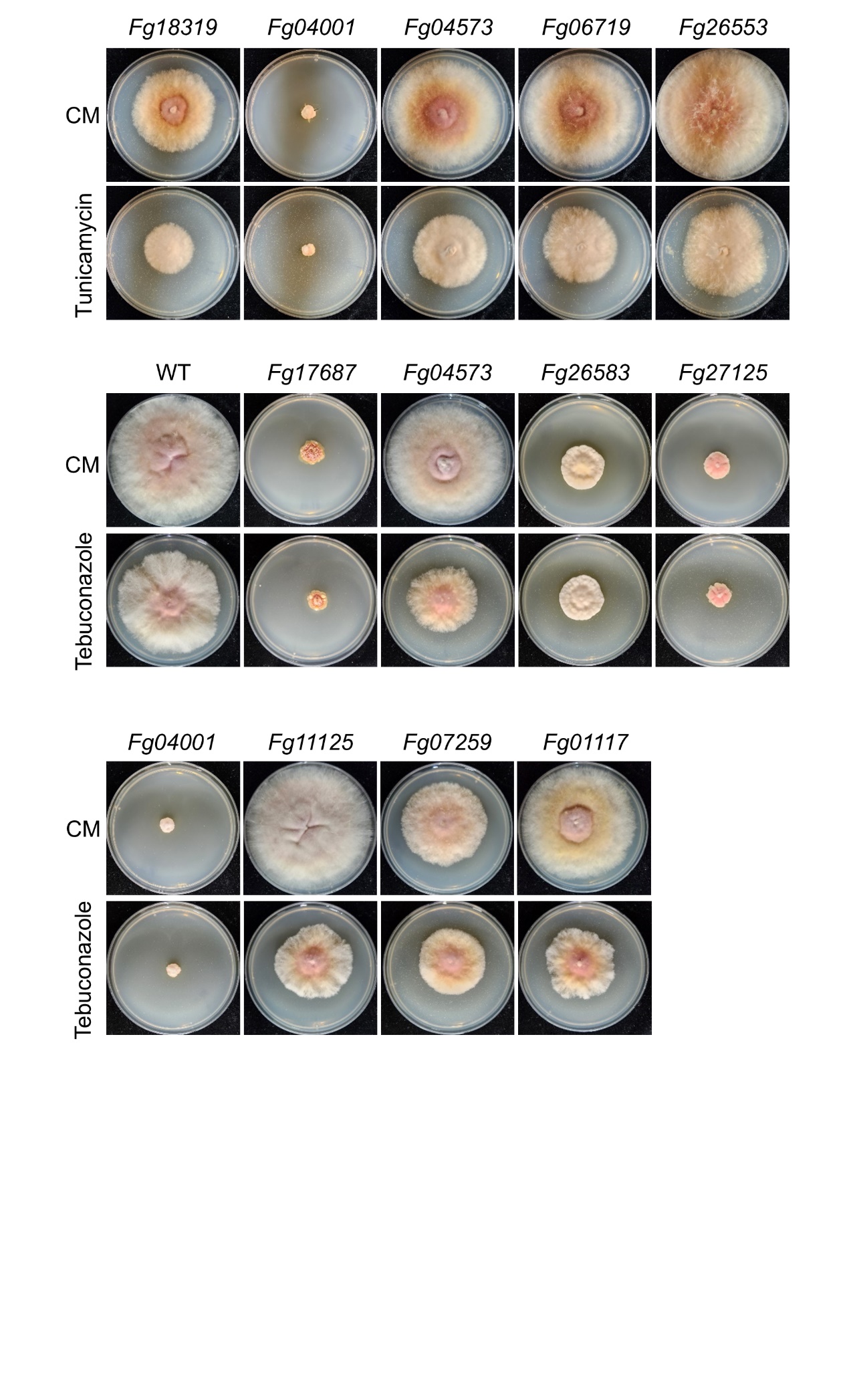
**

**Figure E. Phenotype of deletion mutants under various stress conditions.** The photographs were taken five days after inoculation. H2O2 CM + 7 mM H2O2, Menadione CM + 38 μM Menadione, NaCl CM + 1 M NaCl, KCl CM + 1 M KCl, Sorbitol KCl CM + 1.5 M Sorbitol, FeSO4 CM + 5 mM FeSO4, pH4, pH11, Congo Red CM + 400 mg/L Congo Red , SDS CM + 100 mg/L sodium dodecyl sulfate, Fludioxonil CM + 0.023 mg/L Fludioxonil, Iprodione CM + 8.6 mg/L Iprodione, Benomyl CM + 0.1 mg/L Benomyl, Dimethomorph CM + 2.5 μg/ml Dimethomorph, Caspofungin CM + 0.2 μg/ml Caspofungin, Anidulafungin CM + 0.03 μg/ml Anidulafungin, Micafungin CM + 0.02 μg/ml Micafungin, DTT CM + 10 mM dithiothreitol, Tunicamycin CM + 2.5 μg/ml Tunicamycin, Tebuconazole CM + 0.1 mg/L Tebuconazole.

**
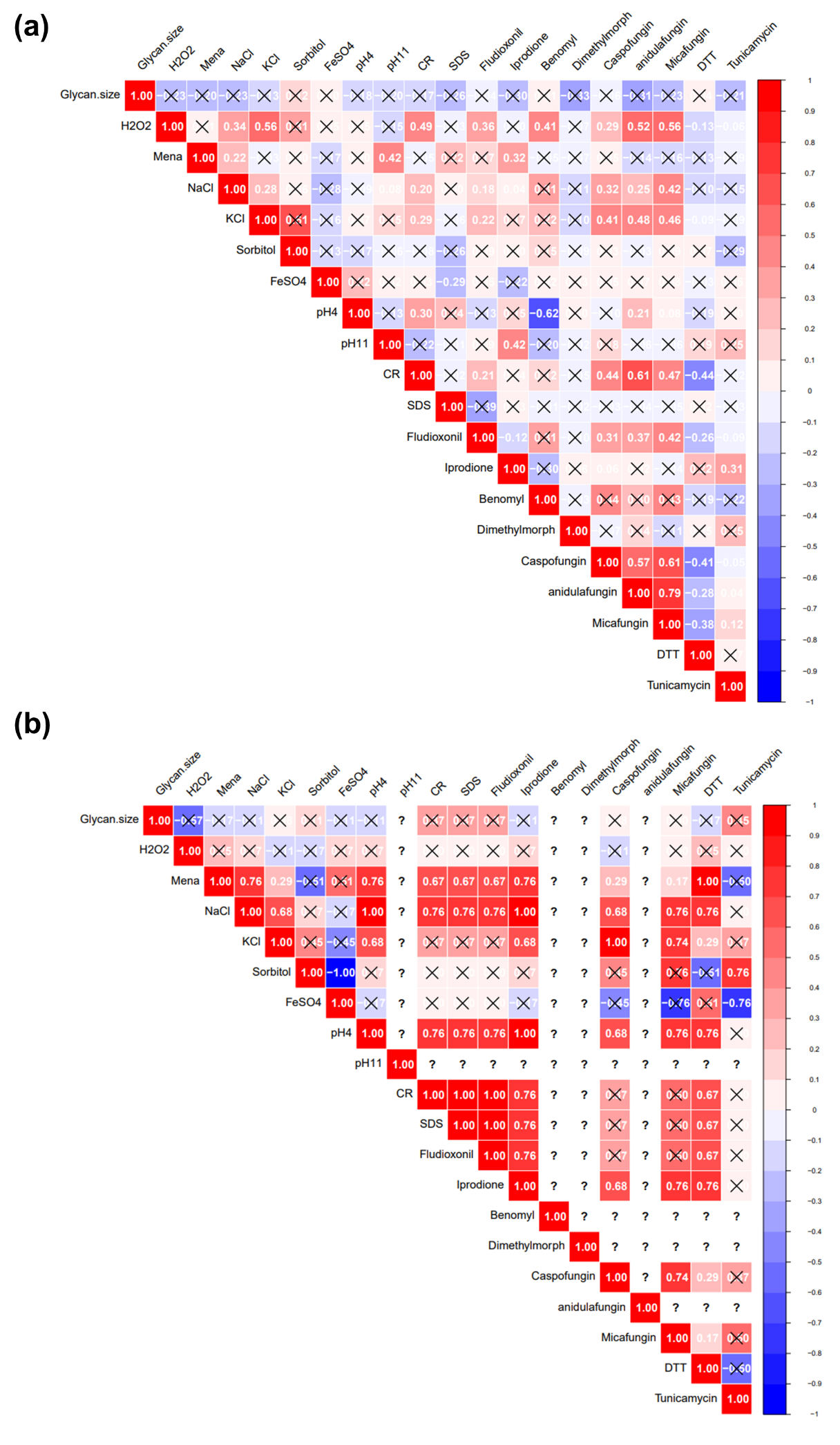
**

**Figure F. Spearman's rank correlation among multiple phenotypes under various stress conditions was calculated for each mutant phenotype. ‘**X’ indicates that there is no significant correlation between the two phenotypes (*P* < 0.05; two-tailed *t*-test). ‘?’ indicates that the correlation between the two phenotypes having zero standard deviation. The size of each glycan was manually determined based on the number of mannose and glucose residues, using the diagrams of the glycosylation pathway in *Saccharomyces cerevisiae* shown in Fig. 2a and 3a. (a) Genes involved in *N-*glycosylation, (b) Genes involved in *O*-glycosylation.

**
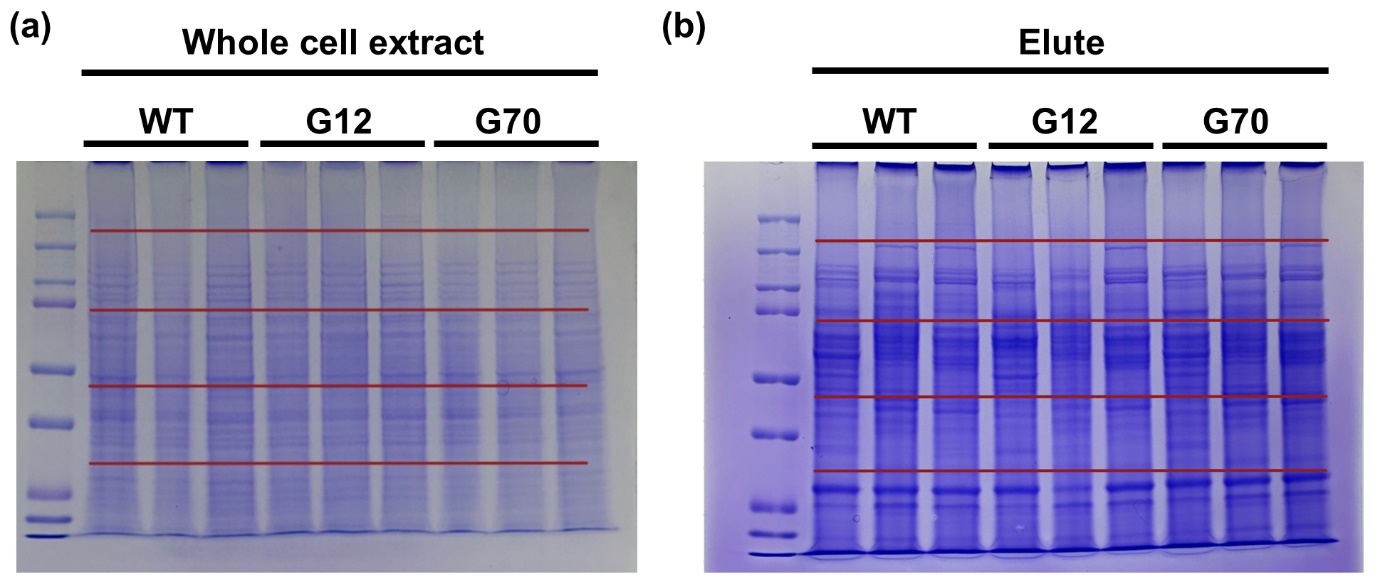
**

**Figure G. Sample preparation for glycoproteomic analysis.** Electrophoretic analysis of whole cell extracts and glycoproteins of wild type, *fg03053*, and *fg26583* deletion mutants. (a) SDS-PAGE showing proteins from whole cell extracts. (b) SDS-PAGE of the purified glycoprotein samples. All samples were loaded in equal volumes and at equal concentrations (1 mg/ml, 100 μl). Red line indicates the gel cutting site for analysis.

**
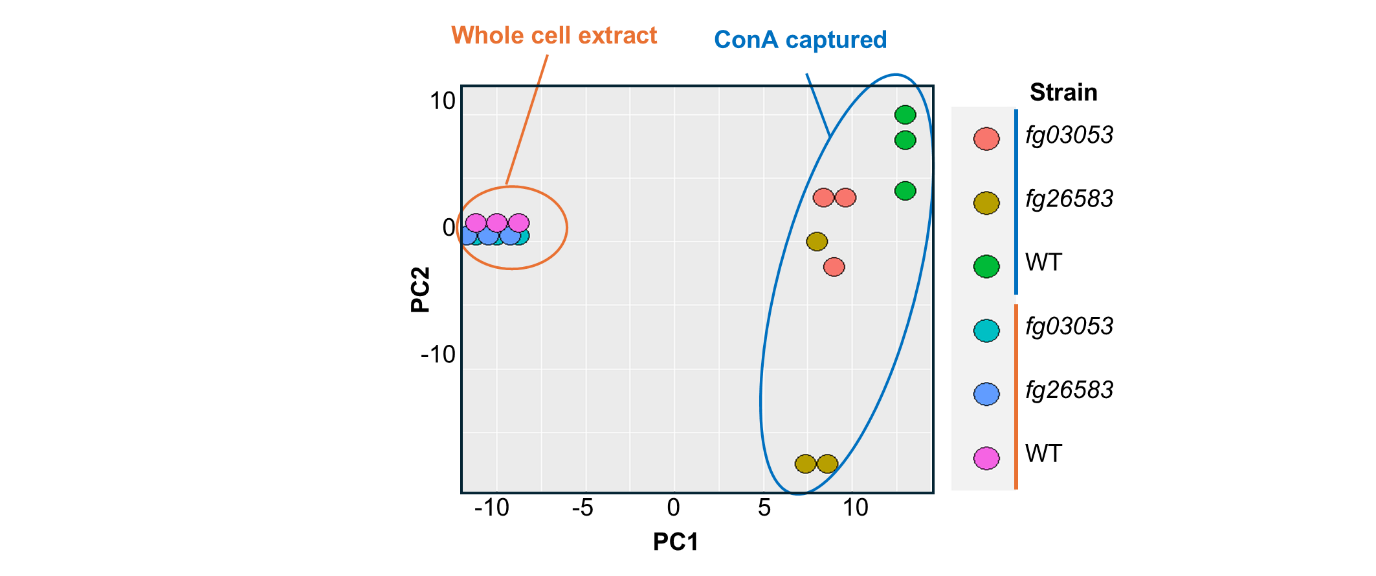
**

**Figure H. PCA analysis for glycoproteomic samples.** The principal component analysis (PCA) was carried out to get an overview of the data to detect batch effects and assess differences between replicates. The retained proteins were identified in three replicates of at least one condition.

**
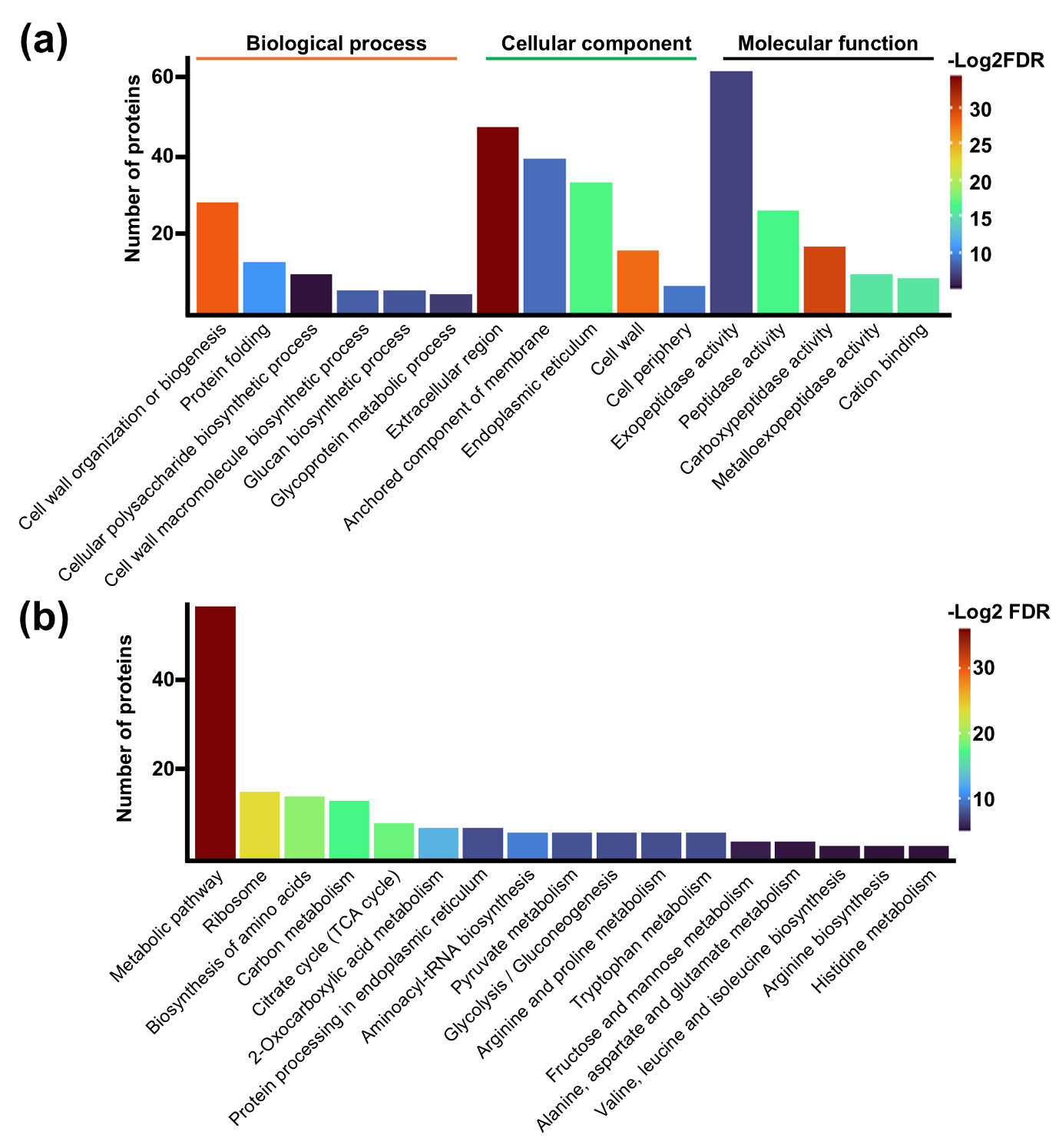
**



**Figure I. Prediction of subcellular localization and functional classification of identified glycoproteins. (**a) Functional classification of identified glycoproteins based on Gene Ontology analysis. (b) KEGG pathway enrichment of glycosylated proteins.

**
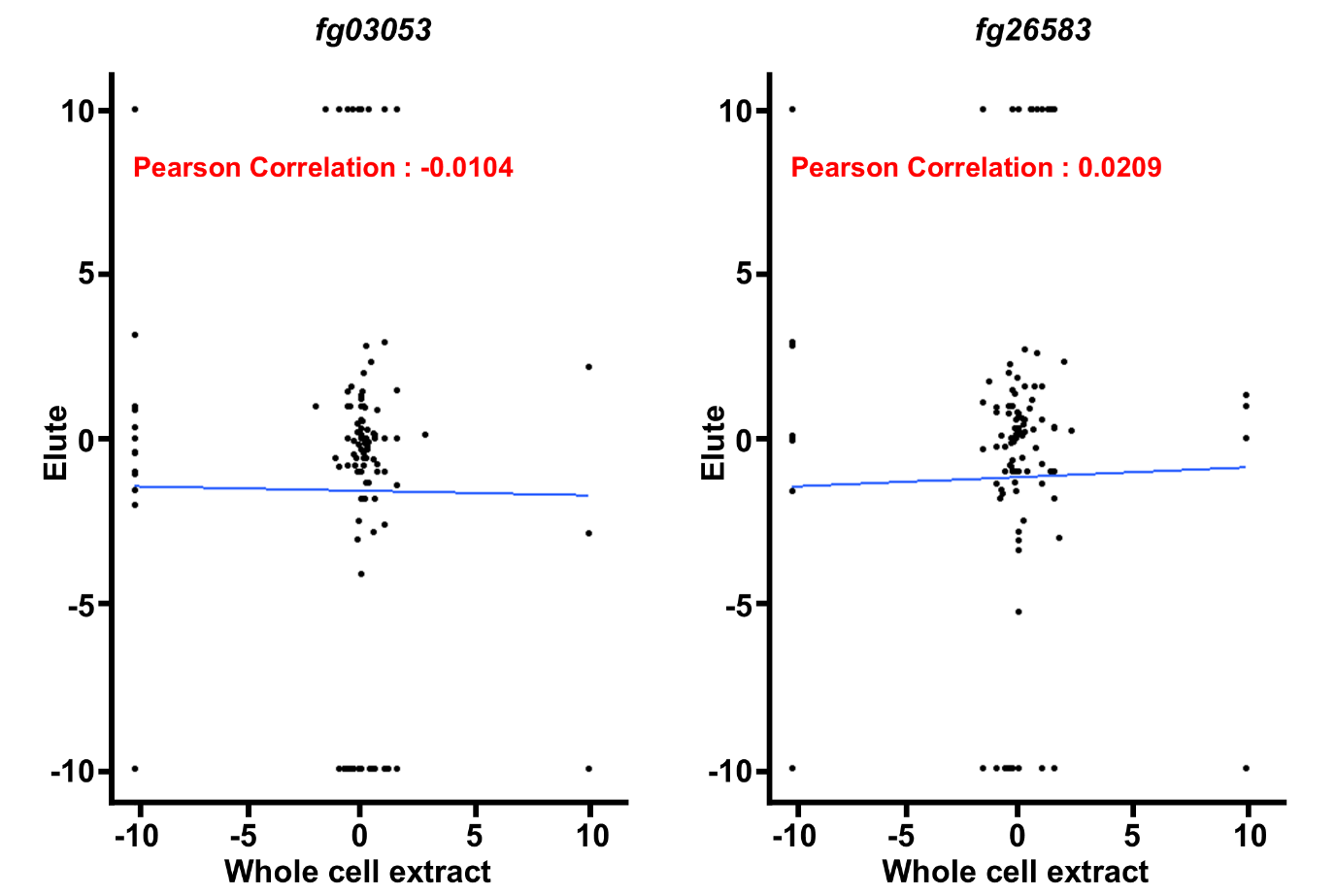
**

**Figure J. Pearson correlation analysis between whole-cell extracts and glycoprotein samples in the wild type, *fg03053*, and *fg26583* deletion mutants.**

**References**

1. Zhang X-W, Jia L-J, Zhang Y, Jiang G, Li X, Zhang D, et al. In Planta Stage-Specific Fungal Gene Profiling Elucidates the Molecular Strategies of *Fusarium graminearum* Growing inside Wheat Coleoptiles  The Plant Cell. 2012;24(12):5159-76. doi: 10.1105/tpc.112.105957.

2. Son H, Lim JY, Lee Y, Lee Y-W. Utilization of a conidia-deficient mutant to study sexual development in *Fusarium graminearum*. PLoS One. 2016;11(5):e0155671.

3. Sikhakolli UR, López-Giráldez F, Li N, Common R, Townsend JP, Trail F. Transcriptome analyses during fruiting body formation in *Fusarium graminearum* and Fusarium verticillioides reflect species life history and ecology. Fungal genetics and biology. 2012;49(8):663-73.

4. Zhao C, Waalwijk C, de Wit PJ, Tang D, van der Lee T. Relocation of genes generates non-conserved chromosomal segments in *Fusarium graminearum* that show distinct and co-regulated gene expression patterns. Bmc Genomics. 2014;15:1-17.
